# Pharmacological evaluation of physcion as a TRPV1 inhibitor with multimodal analgesic efficacy in experimental pain models

**DOI:** 10.1186/s40659-025-00630-5

**Published:** 2025-07-10

**Authors:** Hanbin Chen, Guanghong Li, Lin Deng, Shuli Li, Songqiang Huang, Simon Ming-Yuen Lee, Xiaowei Nie, Jin-Song Bian

**Affiliations:** 1 Department of Pharmacology, Joint Laboratory of Guangdong-Hong Kong Universities for Vascular Homeostasis and Diseases, SUSTech Homeostatic Medicine Institute, School of Medicine, Southern University of Science and Technology, Shenzhen, China; 2 Department of Cardiology, The Eighth Affiliated Hospital of Sun Yat-sen University, Shenzhen, Guangdong China; 3 State Key Laboratory of Quality Research in Chinese Medicine, Institute of Chinese Medical Sciences, University of Macau, Macao, China; 4 School of Biomedical Sciences, Affiliated Hospital of Hunan University, Hunan University, Changsha, Hunan China; 5 Department of Food Science and Nutrition, The Hong Kong Polytechnic University, Hung Hom, Hong Kong China; 6 PolyU-BGI Joint Research Centre for Genomics and Synthetic Biology in Global Ocean Resources, The Hong Kong Polytechnic University, Hung Hom, Hong Kong China; 7 Research Centre for Chinese Medicine Innovation, The Hong Kong Polytechnic University, Hung Hom, Hong Kong China; 8 State Key Laboratory of Chemical Biology and Drug Discovery, The Hong Kong Polytechnic University, Hung Hom, Hong Kong China; 9 Research Institute for Future Food, The Hong Kong Polytechnic University, Hung Hom, Hong Kong China; 10 Research Institute for Smart Ageing, The Hong Kong Polytechnic University, Hung Hom, Hong Kong China; 11 Department of Human Cell Biology and Genetics, School of Medicine, Southern University of Science and Technology, Shenzhen, China

**Keywords:** Physcion, Virtual screening, Molecular dynamics simulation, Aanalgesic, Anti-inflammation

## Abstract

**Background:**

Pain serves as a vital protective mechanism triggered by tissue damage. While NSAIDs and opioids offer relief, their prolonged usage is hindered by adverse effects. Developing analgesics with fewer side effects is crucial for effective pain treatment. The TRPV1 channel is a key target for pain relief, with its inhibitors effectively reducing hyperalgesia in animals. This research utilized virtual screening to identify TRPV1-selective natural compounds for potent analgesic properties.

**Results:**

The physcion exhibited the notable affinity for TRPV1 compared to the compounds examined. After conducting molecular dynamics simulations, physcion emerged as the compound demonstrating the highest binding affinity towards TRPV1, a finding corroborated by calcium imaging, which validated its inhibitory impact. Furthermore, physcion mitigated the stretch number in the acetic acid-induced stretching model, prolonged the latency period in the hot water tail-flick and hot plate assays, and heightened the pain withdrawal threshold lowered by complete Freund’s adjuvant (CFA). Notably, physcion exerted a marked effect in ameliorating bone cancer-induced pain in the hot plate and von Frey tests. Additionally, physcion diminished the levels of inflammatory cytokines and the mRNA expression of both inflammatory and calcium-related genes in the CFA-induced murine model. Furthermore, physcion downregulated the expression of inflammatory genes induced by tumor necrosis factor-α (TNF-α) in RAW264.7 cells. The underlying mechanism potentially involves the suppression of the NF-κB and MAPK signaling cascades.

**Conclusions:**

Our investigation underscores the potential of physcion as a promising candidate for analgesic therapy.

**Supplementary Information:**

The online version contains supplementary material available at 10.1186/s40659-025-00630-5.

## Introduction

Chronic pain is a significant public health concern that impacts millions of individuals globally [1]. Pain disrupts daily routines and significantly diminishes the quality of life for patients. Effective pain management is essential to prevent harm to biological systems and uphold humane principles. Since the revelation of opium’s analgesic properties, opioids have stood as a vital element in pain management strategies [2]. Opioids, akin to other drug classes, present adverse effects like vomiting, dizziness, and, notably, tolerance, potentially leading to addiction, which resulted in the current opioid epidemic [3, 4]. There is an urgent demand for a novel analgesic that offers a broad therapeutic window while minimizing side effects.

The transient receptor potential vanilloid 1 (TRPV1) channel belongs to the vanilloid subfamily of TRP channels and was first recognized as a receptor that senses heat within the pain pathway [5]. As a non-selective cation channel, TRPV1 is crucial for pain perception and can be activated by various stimuli, including capsaicin, heat, and pro-inflammatory substances [6]. Through modulation of the vanilloid-binding pocket, TRPV1 can be either activated or inhibited, resulting in the stabilization of the channel in an open or closed conformation [7]. Capsaicin adopts a “tail-up, head-down” conformation within the vanilloid pocket [8]. Its vanillyl group forms critical hydrogen bonds with T551 (S4) and E571 (S4-S5 linker), while hydrophobic interactions between the aliphatic tail and residues like F544 (S4) facilitate outward movement of the S4-S5 linker. This “pull-and-contact” mechanism displaces the S6 helix, opening the activation gate [8]. In contrast, the antagonist capsazepine binds in a “head-up, tail-down” orientation, with its halogenated headgroup positioned near the extracellular end of S3. This configuration fails to engage the S4-S5 linker effectively, preventing the conformational changes required for pore opening and stabilizing the closed state [9]. This detailed characterization of the distinct ligand binding modes and their respective impacts on channel conformation is crucial for subsequent computational studies. Therefore, the modulation of TRPV1 activity using antagonists offers the potential to relieve pain without the typical side effects linked to traditional opioid analgesics. These opioids are notorious for their addictive nature and other detrimental consequences [10]. Consequently, TRPV1-targeting antagonists have emerged as significant focal points in the development of novel analgesics, particularly for the management of chronic pain [11].

Natural products have been established as a crucial reservoir of bioactive compounds. Due to their diverse structures, natural products play a pivotal role in the modern shift from random drug screening to structured drug development [12]. The combination of virtual screening and natural product libraries can significantly expedite the identification of novel TRPV1 antagonists [13]. This approach facilitates the discovery of potential agents for pain control and allows for the investigation of the pharmacological characteristics of natural products, which are often well-documented for their safety and effectiveness due to their extensive utilization in traditional medicine [14].

Molecular dynamic simulations were employed to analyze docked complexes’ stability and binding free energies to investigate the binding affinity further. We found that five significant components exhibited the highest potency in interacting with TRPV1, with physcion demonstrating the most stable interaction compared to the other four compounds. Calcium imaging studies revealed that physcion inhibited Ca^2+^ influx in HEK293-hTRPV1 cells. Furthermore, physcion exhibited significant analgesic effects in various animal models. This effect is likely attributed to its anti-inflammatory properties by suppressing the NF-κB and MAPK signaling pathways. These findings suggested that physcion was the bioactive compound in rhubarb that was responsible for its analgesic effects and that it held promise as a potential analgesic agent.

## Materials and methods

### Ligand of Preparation

The Natural Product Library for HTS library L6000 containing 4013 small molecules were obtained from TopScience Database (https://www.targetmol.cn/topscience-database). These compounds were converted into PDB format utilizing Gypsum-DL 1.2.1 [15, 16]. Subsequently, the OpenBabel 3.1.1 software was employed to convert the format to PDBQT.

### High throughput virtual screening

**Fig. 1:**
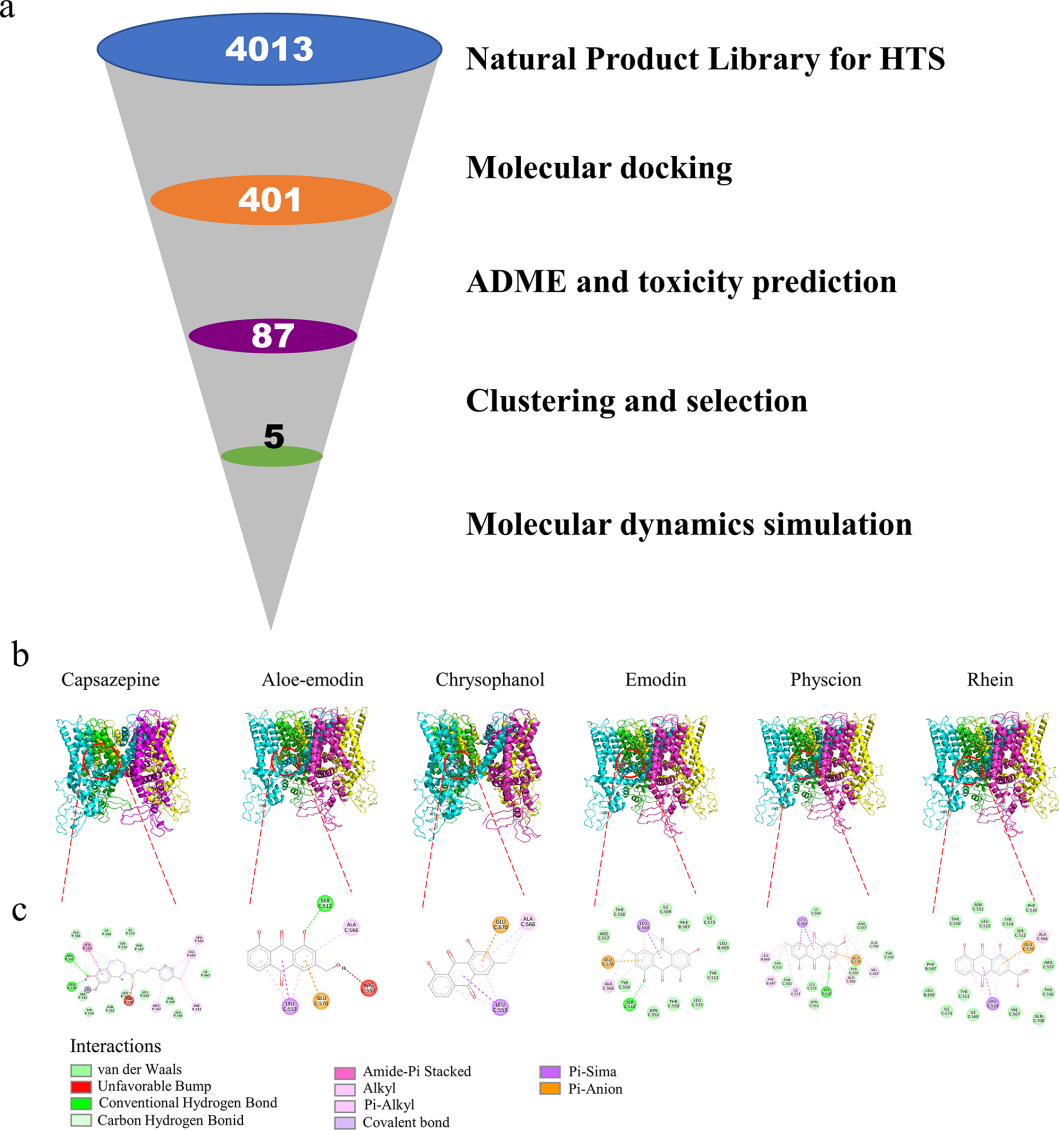
(**a**) The virtual screening workflow. (**b**-**c**) Visualization of the interactions between TRPV1 and capsazepine, aloe-emodin, chrysophanol, emodin, physcion, and rhein

The diagram in Fig. 1 illustrates the bespoke high-throughput virtual screening process. To summarize, 4013 small compounds and TRPV1 underwent docking through Vina-GPU version 2.0 [17]. The definition of the active pocket relied on the localization of the TRPV1 inhibitor capsazepine within the protein structure (PDB ID: 5IS0). The dimensions of the TRPV1 grid box were 30 × 30 × 30 grid points, and its central coordinates were established at x = 109.944, y = 93.765, and z = 104.958. The established criteria encompassed comprehensiveness = 32, energy spectrum = 4, and number of modes = 10. Subsequently, the top decile of scores was selected for further scrutiny. The chosen compound underwent assessment using ADMETlab 3.0 to forecast its absorption, distribution, metabolism, excretion, and toxicity. Moreover, it was screened based on the Lipinski criteria and potential toxicities such as cardiotoxicity, carcinogenicity, neurotoxicity, hematotoxicity, nephrotoxicity, and genotoxicity.

### Molecular dynamics (MD) simulation

The structure of ligand-TRPV1 complexes was submitted to the High-Throughput Simulator of CHARMM-GUI (https://www.charmm-gui.org/) [18]. MD simulations modeled a single ligand bound to the vanilloid pocket of TRPV1. The transmembrane was incorporated using 1-palmitoyl-2-oleoyl-sn-glycero-3-phosphocholine (POPC) according to the methodology [19]. The water model used was TIP3P (as specified in CHARMM-GUI’s default protocol for CHARMM36m forcefield). Water and 150 ml of NaCl solution were introduced into the system to maintain ion balance. The next step involved the energy minimization process, which consisted of 5 × 10^5^ steps. After this, the system underwent equilibration in the canonical ensemble (NVT) and the isothermal-isobaric (NPT) ensemble for 1.2 × 10^6^ steps. Following the equilibration steps, a 100 ns molecular dynamics (MD) simulation was carried out utilizing the CHARMM36m forcefield through GROMACS 2022.6 [20]. Utilizing various analytical tools and methodologies, such as the root mean square deviation (RMSD) of TRPV1 and its ligands, the radius of gyration (Rg), hydrogen bond analysis, solvent-accessible surface area (SASA) calculations, principal component analysis (PCA), and free energy landscape (FEL) assessment, we assessed the binding stability between TRPV1 and the ligands. The 100 ns trajectory was segmented into 200 frames, each representing the binding status of ligands to TRPV1 residues at every 0.5 ns. Protein-Ligand Interaction Profiler (PLIP, version 2.3.0) was used to analyze the interaction between TRPV1 and ligands [21]. Using an in-house Python script, visualize the interactions between TRPV1 residues and ligands throughout a 100 ns simulation. The binding free energy was evaluated through the MMPBSA method employing the gmx_MMPBSA tool (https://github.com/Jerkwin/gmxtool/tree/master/gmx_mmpbsa, accessed on 4 April 2022) [22, 23].

### Method validation

In order to validate the robustness of our Vina-GPU screening outcomes, we conducted molecular docking via AlphaFold3 utilizing Protenix Server. The protein sequence of TRPV1 (Uniport ID: O35433) was entered into the designated interface, and the Simplified Molecular Input Line Entry System (SMILES) of physcion (CAS: 521-61-9) was submitted for molecular docking. The active site was defined as the complete protein, and conformations were organized based on their docking scores. Subsequently, the most favorable conformation was chosen for binding mode assessment. Ultimately, the results were scrutinized and visualized using PyMOL (version 3.0.3) [24] and Discovery Studio Visualizer (version 21.1).

Regarding the validation of the MD simulation workflow, both capsaicin and capsazepine were subjected to docking with TRPV1 via Vina-GPU 2.0. Subsequently, the resulting complexes underwent MD simulations using consistent parameters as detailed earlier. A comparative analysis was carried out between these results and existing literature for validation purposes.

### Materials

Physcion, capsaicin, chloroform, isopropanol, and ethanol were sourced from Shanghai Macklin Biochemical Technology in Shanghai, China. Complete Freund’s adjuvant (CFA) and Fluo-4 were provided by Sigma-Aldrich (Saint Louis, USA), while acetic acid was obtained from Shanghai Aladdin Biochemical Technology in Shanghai, China. Capsazepine (CPZ) and normal saline were acquired from Techisun Bio-technology in Shenzhen, China. Penicillin/streptomycin was supplied from Proteintech Group, Inc. (Wuhan, China). RNA extraction reagent RNAiso Plus was purchased from Takara Biomedical Technology (Dalian, China). cDNA Synthesis Kit and qPCR SYBR Green were supplied from Yeasen Biotechnology (Shanghai, China). Fetal bovine serum (FBS) was provided from ExCell Bio (Shanghai, China). Dulbecco’s modified Eagle medium with high glucose (4.5 g/L) and RPMI1640 medium were obtained from Gibco-BRL in Gaithersburg, USA. Mouse interleukin-1 beta (IL-1β), interleukin-6 (IL-6), and tumor necrosis factor-alpha (TNF-α) Valukine ELISA were provided from Novus Biologicals (Littleton, USA). The nitric oxide (NO) detection kit, radioimmunoprecipitation assay lysis buffer (RIPA) lysis buffer, protease inhibitor phenylmethanesulfonyl fluoride (PMSF), protease and phosphatase inhibitor cocktail, BCA protein assay kit, QuickBlock™ Protein-Free Blocking Buffer for Western Blot and stripping buffer were sourced from Beyotime Biotechnology (Nantong, China). Polyvinylidene difluoride (PVDF) membrane and Immobilon Western HRP substrate were provided from Millipore (Burlington, USA). Phospho-IκB Alpha (Ser32/36) antibody, IκB Alpha antibody, Phospho-NF-κB p65 (Ser468) antibody, NF-κB p65 antibody, Phospho-JNK (Tyr185) antibody, JNK antibody, Phospho-p38 MAPK (Thr180/Tyr182) antibody, p38 MAPK antibody, Phospho-ERK1/2 (Thr202/Tyr204) antibody, ERK1/2 antibody, glyceraldehyde 3-phosphate dehydrogenase (GAPDH) antibody, β-actin antibody, β-Tubulin and Goat Anti-Rabbit IgG (H + L) were obtained from Proteintech Group, Inc. (Wuhan, China).

### Calcium imaging

The human embryonic kidney (HEK293) cells were cultured in DMEM medium supplemented with 10% fetal bovine serum and 1% penicillin-streptomycin. To develop human TRPV1-expressing HEK293 cells, the Lipo8000TM Transfection Reagent was utilized. A plasmid DNA containing the human TRPV1 protein (NM_018727.5, IGE Biotechnology, Guangzhou, China) at a concentration of 2.5 µg was transfected into the HEK293 cells. Following cell seeding in a 12-well plate, they were incubated at 37 ℃ and 5% CO_2_ for 24 h. Various concentrations of physcion were then added and incubated for 4 h. Subsequently, the cells were stained with 2 µM Fluo-4/AM, dissolved in HEPES buffer, and incubated in darkness for 30 min. After incubation, the cells underwent three washes with HEPES buffer. The TRPV1 agonist capsaicin was applied at a concentration of 1 µM to induce calcium influx in all calcium imaging experiments. The measurement of calcium fluorescence was promptly carried out using the cellSens imaging system integrated into an IX73 microscope produced by Olympus Co. in Tokyo, Japan [25]. This assessment’s excitation and emission wavelengths were set at 494 nm and 516 nm, respectively. Temporal fluorescence alterations were quantified using ImageJ (version 1.48).

### Animals

The study employed male C57BL/6J mice (8-10-week-old) sourced from GemPharmatech (Nanjing, China). Consistency in utilizing male mice was observed throughout all experiments. The allocation of mice to experimental groups was randomized. During the research duration, the mice resided in a controlled environment following a 12-hour light-dark cycle, with light onset at 7 a.m. The ambient temperature was kept within the 23–25 °C range to ensure animal comfort. They were provided unrestricted access to both water and standard laboratory chow. Housing occurred in a specific pathogen-free (SPF) facility to maintain their health and welfare. All animal procedures were carried out using protocols approved by the Institutional Animal Care and Use Committee of the Southern University of Science and Technology. Adherence to the prescribed protocols and guidelines concerning the ethical treatment of animals was rigorously upheld throughout the study period.

### Animal behavior

#### Body temperature

Due to the impact of TRPV1 inhibitors on thermoregulation, 6 mice were individually identified. Subsequently, the rectal temperature of each mouse was initially assessed, and then following the administration of physcion, the temperatures were recorded again. Rectal temperatures were recorded at baseline (0 h) and at 1, 2, 4, 5, 8, 12, and 24 h post-administration. The temperature differentials pre- and post-administration were then calculated.

#### Acetic acid-induced stretching

Initially, 32 mice were distributed into four groups: the control group received pretreatment with saline only, without intraperitoneal injection (i.p.) of 0.6% acetic acid; the vehicle group was pretreated with saline and received i.p. infusion of 0.6% acetic acid. The remaining two groups were pretreated with CPZ (20 mg/kg) or physcion (20 mg/kg) followed by i.p. injection of 0.6% acetic acid. The administration of drugs occurred 1 h before the 0.6% acetic acid treatment. Each mouse was placed in a Polymethyl methacrylate (PMMA) observation chamber allowing a 20-minute acclimatization period. Observations were initiated 5 min post the acetic acid injection. A stretch was defined operationally as an abdominal contraction followed by hind limb extension [26]. The number of stretches was documented over 20 min.

#### Hot water tail-flick test

To evaluate the analgesic efficacy of physcion (20 mg/kg, i.p.), we conducted a tail-immersion test using a controlled water bath system. Sixteen age-matched mice were randomly allocated into two experimental cohorts: saline-treated controls (*n* = 8) and physcion-treated (20 mg/kg, *n* = 8). Following a 7-day acclimatization period, test compounds were administered intraperitoneally 1 h prior to behavioral assessment. During testing, animals were gently restrained in breathable cotton sheaths with their distal tail segment (2–3 cm) immersed in a 50 °C water bath. Tail-withdrawal latency was recorded from immersion onset to the first rapid flick response, with a 12-second cutoff established to prevent tissue damage. Each animal underwent three consecutive trials at 4-min intervals, during which subjects were returned to their home cages for recovery. Mean latency values were calculated from three technical replicates [27].

#### Hot plate test

The effects of physcion (20 mg/kg; i.p.) and CPZ (20 mg/kg; i.p.) were evaluated using a hot plate thermal nociceptive test, utilizing temperature parameters and experimental settings established in previous studies [28]. Twenty-four C57BL/6 mice (8-week-old, male) were randomly assigned to three treatment groups (*n* = 8/group): (1) saline control, (2) CPZ (20 mg/kg), and (3) physcion (20 mg/kg). Following a 3-day habituation period, test compounds were administered 60 min prior to behavioral testing conducted during the light phase (9:00–12:00).

Nociceptive responses were quantified as latency to either hind paw licking, jumping, or reaching the safety cutoff (25 s), with immediate removal upon response manifestation to prevent tissue injury. Baseline measurements were obtained on two consecutive days pre-treatment: mice were placed in a plexiglass cylinder (20 cm diameter) on the heated surface, with two measurements separated by 1 h intervals to avoid thermal sensitization. The mean baseline latency served as individual reference values.

#### CFA-induced inflammatory pain

In 1986, Larson et al. established the CFA-induced inflammatory pain model, a widely recognized paradigm of persistent inflammatory hyperalgesia [29]. The standard procedure entails injecting 20 µl of CFA into the plantar surface of the right hind paw to induce inflammation. The mice were randomly divided into seven groups: Group 1, the control group receiving normal saline; Group 2, the group with CFA-induced inflammation; Group 3, the CPZ group treated with CFA; and Groups 4 to 6, the physcion treatment groups (administered at doses of 10, 15, and 20 mg/kg) in conjunction with CFA administration. Group 7 was administered physcion (20 mg/kg) without CFA induction. Test compounds were administered 60 min prior to behavioral testing conducted during the light phase.

Mechanical allodynia, assessed through the von Frey test, was evaluated pre- and post-CFA injection at 7 days. The mice’s mechanical response threshold of escape behavior was assessed using a series of ascending von Frey filaments (North Coast Medical Inc., CA, USA), starting with the smallest filament with a bending force of 0.16 g. Before the experimentation, the mice were acclimatized individually in 6 × 6 cm enclosures on a raised wire grid for 30 min each day spanning three consecutive days. On the experimental day, following a 30-minute acclimation period in the testing environment, the mice’s hind paws were stimulated with von Frey filaments of logarithmically increasing stiffness (ranging from 0.16 to 2.56 g) applied perpendicularly to the central plantar surface. A positive response was noted when the mouse reacted to the filament, with the corresponding force recorded; conversely, a lack of response indicated a negative result, leading to the application of the next larger filament. The mechanical withdrawal threshold was measured in grams (g) using the specified formula [30], representing the pressure exerted by the von Frey filament that triggered a response from the animal.

#### Bone cancer pain model

The bone cancer pain model was established following the methodology outlined in a previous study [31]. The murine Lewis lung carcinoma cell line LLC1 (ATCC^®^ CRL-1642) was procured from the American Type Culture Collection (ATCC). These cells were nurtured in Dulbecco’s modified Eagle medium with high glucose, supplemented with 10% fetal bovine serum and 1% penicillin/streptomycin solution, and maintained at 37 °C in a 5% CO_2_ environment.

To create the bone cancer pain model, the LLC1 cell line was disassociated through 0.05% trypsin. The cells were then suspended in phosphate-buffered saline (PBS) at 5 × 10^7^ cells/mL. Subsequently, mice were anesthetized with 2.5% isoflurane, and a superficial incision of 0.5 to 1 cm was performed near the knee to expose the patellar ligament. A 25-gauge needle was then inserted into the femoral cavity at the intercondylar notch of the left femur. This needle was later exchanged with a 10 µL microinjection syringe containing a 4 µL suspension of tumor cells (2 × 10^5^) and 2 µL of an absorbable gelatin sponge solution to seal the injection site. The syringe contents were slowly injected into the femoral cavity over 2 min. The external injection site was sealed with silicone adhesive to prevent tumor cell leakage from the bone cavity (Kwik-Sil, World Precision Instruments). Animals that encountered unsuccessful injections or displayed compromised mobility post-surgery were excluded from the research study. Subsequently, to assess the protective impact against bone cancer pain, either physcion or CPZ (20 mg/kg, administered intraperitoneally) was given on day 7 after inoculation. The evaluation of bone pain involved thermal and mechanical pain assessments through two standardized behavioral tests. Test compounds were administered 60 min prior to behavioral testing conducted during the light phase. For mechanical allodynia assessment, the von Frey test was performed by applying calibrated filaments (0.16–2.56 g force range) to the plantar surface of the ipsilateral hind paw. Mice were placed in individual plexiglass chambers on an elevated mesh platform and allowed to acclimate for 30 min prior to testing. Each filament was applied vertically to the mid-plantar area with sufficient force to cause slight bending, maintained for 3 s. A positive response was defined as rapid paw withdrawal or licking. The mechanical withdrawal threshold was measured in grams (g) using the specified formula [30] Thermal hyperalgesia was evaluated using the hot plate test (55 °C), with the latency to hind paw licking or jumping recorded as the nociceptive endpoint.

#### Assessment of inflammatory cytokines by enzyme-linked immunosorbent assay (ELISA)

After 7 days post-CFA injection, behavioral assessments were carried out to validate the successful establishment of the models. Following this, the mice were humanely euthanized through cervical dislocation. Blood samples were obtained via cardiac puncture, left to coagulate at room temperature for 30 min, and then centrifuged at 2000 × g for 10 min at room temperature to acquire the plasma supernatant. ELISA protocols were executed following the manufacturer’s instructions, and a distinct standard curve was established for each trial.

#### Assessment of mRNA expression of dorsal root ganglia (DRG)

Following the administration of CFA, behavior tests were conducted seven days later to confirm the successful establishment of the models. Subsequently, different groups of the mice underwent euthanasia through cervical dislocation, with dissections carried out under chilled conditions. Physcion administration commenced 24 h after CFA injection and continued daily until tissue collection on day 7 post-CFA injection. The L4–L6 DRG were then carefully isolated, ensuring thorough removal of connective tissues. Post-dissection, the DRG tissues were promptly frozen and stored at -80 ℃. RNA extraction was performed using RNAiso Plus, with approximately 1 µg of RNA undergoing reverse transcription to cDNA using a reverse transcription kit per the manufacturer’s guidelines. Subsequent qPCR analysis was conducted utilizing the SYBR Green PCR kit. The primer sequences designed for this study can be found in the supplementary materials. PCR was executed starting with an initial denaturation step at 95 °C for 30 s, followed by 40 amplification cycles of 60 °C for 30 s, 72 °C for 30 s, and 95 °C for 10 s, and 65 °C for 5 s each. Data normalization was conducted relative to the mRNA expression of 18 S rRNA, which was used as the internal control. The quantification and presentation of results for the target gene were performed employing the 2^−ΔΔCT^ method [32].

#### Cell culture

The RAW264.7 macrophage cell line, obtained from ATCC under the designation ATCC TIB-71, was cultured in RPMI1640 medium augmented with 10% (v/v) fetal bovine serum (FBS) and 1% (v/v) penicillin-streptomycin at 37 °C with 5% CO₂ in a humidified incubator.

#### Assessment of mRNA expression of RAW264.7

The RAW264.7 cells were seeded in a 6-well plate following a 24-hour incubation period to facilitate adherence and growth. Sequentially, the cells underwent treatment with varying concentrations of physcion, ranging from 10 to 40 µM, and were co-cultured for 1 h. Subsequently, TNF-α was introduced to achieve a final concentration of 10 ng/mL, and the cells were further incubated for 4 h. Cell mRNA was then isolated utilizing RNAiso plus, following the protocol’s procedures detailed in Sect. 2.9. The primer sequences can be found in the supplementary material.

#### Western blotting

After co-incubation of RAW264.7 cells with 40 µM of CPZ or physcion for 1 h, TNF-α was introduced to achieve a final 10 ng/mL concentration. Subsequently, the cells were co-cultured for an additional hour. Following this, the cells underwent three washes with PBS and were then lysed using RIPA buffer containing 1% PMSF and 1% phosphatase inhibitor cocktail for 20 min on ice. The resulting lysate underwent centrifugation at 13,000xg for 15 min at 4 °C. Protein concentrations in the supernatant were quantified using the BCA assay to ensure uniformity across all experimental sets. Subsequently, the samples were subjected to electrophoresis on a 10% SDS-PAGE gel and transferred to PVDF membranes, which were then blocked using QuickBlock blocking buffer. Immunoblot analysis was performed by incubating the samples with antibodies targeting p-IκBα (1:5000), IκBα (1:5000), p-p65 (1:2000), p65 (1:1000)), p-p38 (1:1000), p38 (1:2000), p-ERK1/2 (1:1000), ERK1/2 (1:2000), p-JNK (1:1000), and JNK (1:3000). Internal loading controls such as GAPDH (1:50000), β-actin (1:20000), and β-Tubulin (1:20000) were employed for normalization.

#### Quantification and statistical analysis

Data and statistical analysis utilized GraphPad Prism version 8.3. The results were presented as the mean ± standard deviation (SD), and the figures indicated significance values. Comprehensive statistical details for the experiments are available in the figure captions of each respective figure. The unpaired Student’s t-test was applied to compare the means between the two groups; a one-way ANOVA with Bonferroni post hoc correction for multiple comparisons was conducted for comparisons involving more than two groups.

## Results

### Identification of potential therapeutic target

The objective of this study is to identify novel TRPV1 inhibitors and investigate their analgesic effects. The virtual screening workflow is illustrated in Fig. 1a. Natural Product Library for HTS L6000 comprising 4,013 phytochemicals was subjected to Vina-GPU 2.0 for docking against TRPV1. The top 10% of compounds were further evaluated for ADME and toxicity predictions. A total of 87 compounds were obtained and subsequently clustered for visual investigation. Ultimately, five compounds were selected: Aloe-emodin, chrysophanol, emodin, physcion, and rhein.

TYR511, LEU515, LEU547, THR550, ARG557 and GLU570 were the key residue for inhibition of TRPV1 [33, 34]. Aloe-emodin exhibited a pi-anion interaction with GLU570 while demonstrating an unfavorable interaction with ARG557 (Fig. 1b-c). Furthermore, chrysophanol exclusively interacted with GLU570 of TRPV1. Additionally, emodin displayed multiple interactions with TRPV1, involving TYR511, LEU515, THR550, ARG557, and GLU570. The docking analysis demonstrated that capsazepine engaged in multiple interactions with TRPV1, specifically with TYR511, LEU515, ARG557, and GLU570 (Fig. 1b). Similarly, physcion and rhein exhibited identical interactions with TRPV1 as emodin. The results revealed that emodin, physcion, and rhein demonstrated the most significant potency in inhibiting TRPV1. Subsequently, these five compounds underwent molecular dynamics simulation.

We employed AlphaFold3 for full-length TRPV1 modeling and utilized AlphaFold3 for physcion docking with TRPV1 (Figure S1). The results were consistent with those obtained using Vina-GPU. Physcion bound to the vanilloid-binding pocket of TRPV1, interacting with the same amino acids, including TYR511, SER512, LEU515, THR550, ASN551, LEU553, ILE569, GLU570, ILE573, and others. This indicated that the physcion-TRPV1 complex predicted by AlphaFold3 was highly consistent with our original docking pose. The cross-method agreement between these approaches confirmed the reliability of our screening results.

### Molecular dynamics simulation of the five compounds with TRPV1

#### Qualitative overview of MD simulation

**Fig. 2:**
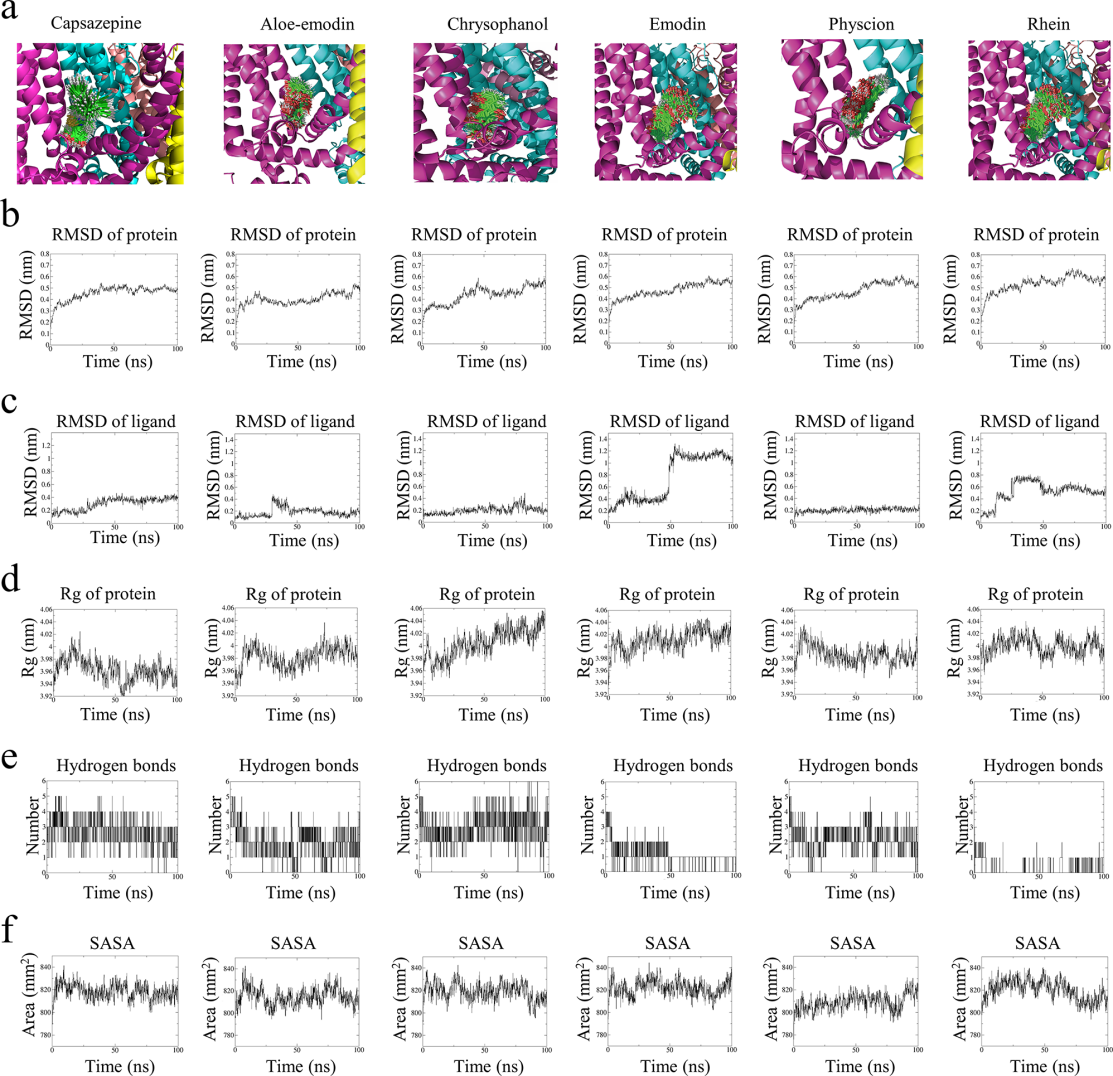
The MD simulation parameters of TRPV1 and capsazepine, aloe-emodin, chrysophanol, emodin, physcion, and rhein. (**a**) The superimposed trajectories of protein-ligand. (**b**) The RMSD values of TRPV1. (**c**) The RMSD values of aloe-emodin, chrysophanol, emodin, physcion, and rhein. (**d**) The Rg values of TRPV1. (**e**) The hydrogen bonds number formed by TRPV1 and ligands. (**f**) The SASA values of TRPV1

Figure 2a provided a qualitative overview of the positional sampling of the five ligands within the TRPV1 binding pocket during the 100-ns MD simulations. Visual analysis of the trajectories indicates varied degrees of conformational stability: emodin and rhein displayed higher positional fluctuations, whereas capsazepine and physcion maintained a relatively confined motion within the pocket, with aloe-emodin and chrysophanol exhibiting intermediate behavior.

#### Root mean square deviation (RMSD) of protein

The RMSD value can be utilized to indicate the molecular motion dynamics throughout the simulation period. Upon binding with ligands, resulting in the conformational change of TRPV1 (Fig. 2b), the RMSD of TRPV1 continuously increased. Significant fluctuations were observed following the binding of chrysophanol or rhein with TRPV1. Aloe-emodin exhibited lower fluctuations but did not stabilize after 100 ns simulation. The RMSD of TRPV1 remained stable over the entire 100 ns simulation period when TRPV1 interacting with capsazepine. Conversely, after the binding of emodin or physcion with TRPV1, the RMSD of TRPV1 appeared more stable compared to the other small molecules.

#### RMSD of ligand

Upon binding with TRPV1, emodin exhibited significant fluctuations at 50 ns. In contrast, rhein and aloe-emodin showed fluctuations around 20 ns, gradually increasing before stabilizing thereafter (Fig. 2c). Upon binding to TRPV1, capsazepine exhibited fluctuations around 20 ns, gradually increased, and eventually stabilized thereafter. The most stable behaviors were observed in physcion and chrysophanol. In comparison, chrysophanol displayed a minor fluctuation of around 75 ns.

#### Radius of gyration (Rg)

Rg refers to the compactness of proteins, where a lower value of Rg indicates a tighter protein structure. In our results, it is observed that upon binding with chrysophanol or emodin, the Rg values of the protein consistently increased (Fig. 2d). Furthermore, upon the binding of aloe-emodin or rhein with TRPV1, the Rg values exhibited fluctuations over the simulation time. In contrast, the Rg value decreased consistently after physcion bound with TRPV1. The average Rg value for capsazepine was calculated to be 3.96, suggesting that its interaction with TRPV1 resulted in a more compact structure of TRPV1. The mean Rg value for physcion was 3.99, placing it third after aloe-emodin’s 3.98 and capsazepine’s 3.96, smaller than chrysophanol’s 4.00, emodin’s 4.01, and rhein’s 4.00.

#### Hydrogen bonds

These five compounds exhibited varying numbers of hydrogen bonds upon interaction with TRPV1. The average intermolecular hydrogen bonds formed between aloe-emodin-TRPV1, chrysophanol-TRPV1, emodin-TRPV1, physcion-TRPV1, rhein-TRPV1, and capsazepine-TRPV1 were 1.941, 2.891, 1.116, 2.225, 0.243, and 2.66, respectively (Fig. 2e). Notably, when physcion bound to TRPV1, it formed the third highest number of hydrogen bonds, which was lower than that of chrysophanol and capsazepine. This finding suggested that the binding of chrysophanol or physcion to TRPV1 resulted in a more stable interaction than that of the other compounds.

#### Solvent accessible surface area (SASA)

SASA analysis enables the quantification of the proportion of protein surface accessible to solvent molecules and facilitates the investigation of interactions between complexes and solvents during MD simulations. A decreased SASA value signifies enhanced thermodynamic stability. The average SASA values for aloe-emodin-TRPV1, chrysophanol-TRPV1, emodin-TRPV1, physcion-TRPV1, rhein-TRPV1, and capsazepine-TRPV1 were 814.79 nm^2^, 818.84 nm^2^, 821.89 nm^2^, 809.04 nm^2^, 820.20 nm^2^, and 818.34 nm^2^, respectively (Fig. 2f). These findings suggested that physcion bound to TRPV1, leading to a more compact conformation of the TRPV1 protein.

#### Principal component analysis (PCA)

**Fig. 3:**
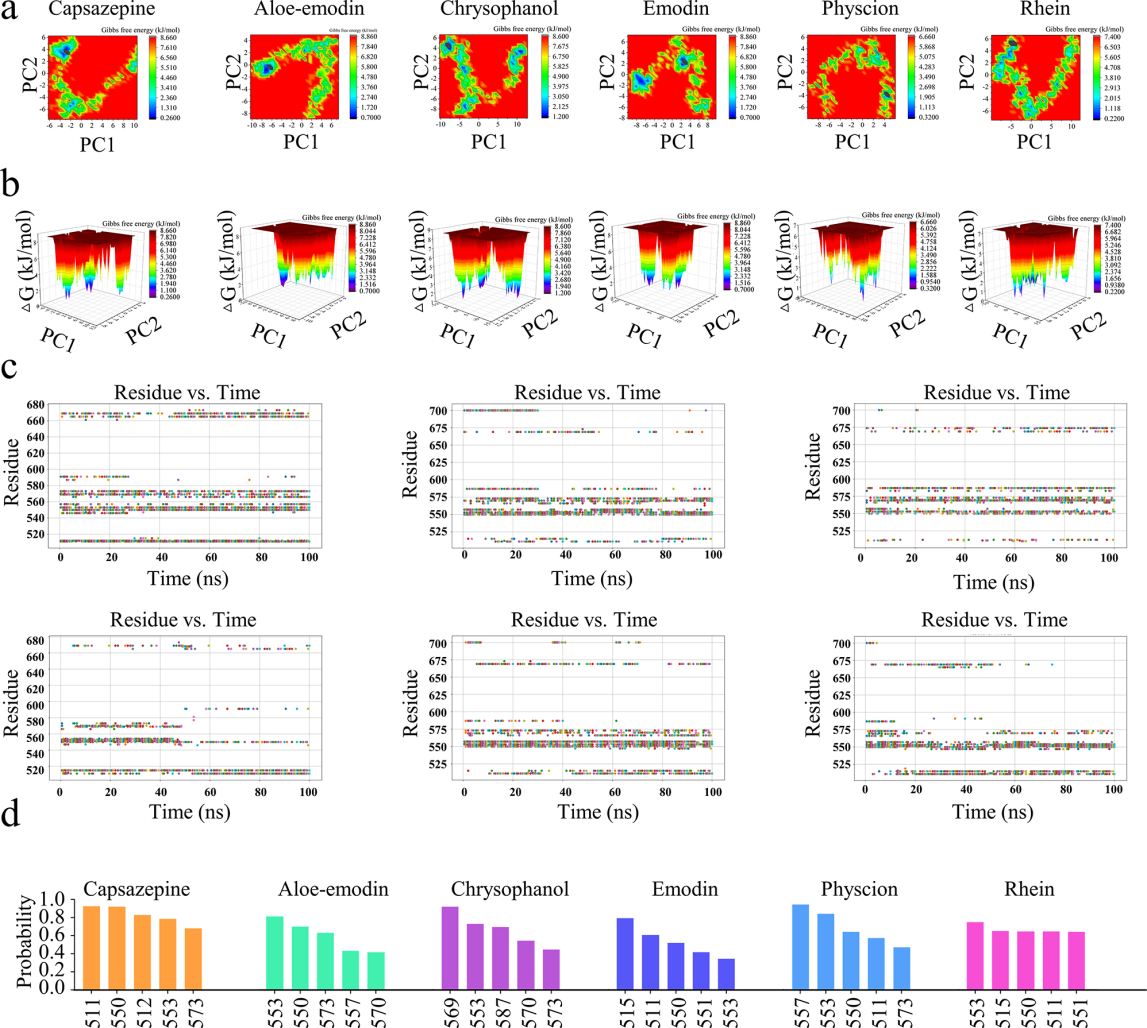
Illustration of the molecular dynamics (MD) simulation profiles involving capsazepine-TRPV1, aloe-emodin-TRPV1, chrysophanol-TRPV1, emodin-TRPV1, physcion-TRPV1, and rhein-TRPV1 complexes. (**a**) Depiction of the two-dimensional protein conformational alterations throughout the simulation utilizing principal components PC1 and PC2 for the protein-ligand complexes. (**b**) Visualization of the Gibbs free energy landscapes of the protein-ligand complexes through three-dimensional representations of the protein conformational modifications. (**c**) Examine the interactions between TRPV1 residues and ligands throughout a 100 ns simulation. The 100 ns trajectory was segmented into 200 frames, each representing the binding status of ligands to TRPV1 residues at every 0.5 ns. Distinct colors denote each 0.5 nanoseconds interval (Panels are arranged in sequential order from left to right and top to bottom). (**d**) Analysis of the interaction probabilities between the top 5 TRPV1 residues and ligands

Principal Component Analysis (PCA) was applied to the displacements of Cα atoms to map the conformational landscapes and identify prominent alterations in collective motions during MD simulations [35]. The projections onto the first two principal components (PCs), specifically PC1 and PC2, represent dominant modes of motion and were used to visualize conformational changes. The results revealed that aloe-emodin, emodin, and physcion clustered in similar regions of the PCA subspace, reflecting comparable conformational dynamics upon TRPV1 binding. Furthermore, principal component analysis (PCA) indicated that capsazepine, chrysophanol, and rhein grouped closely in the PCA subspace and explored a wider conformational space (Fig. 3a), indicating greater conformational flexibility and distinct cluster patterns. Conversely, the TRPV1-physcion complex sampled a narrower region of the PCA subspace, reflecting restricted conformational dynamics and enhanced stability.

#### Gibbs free energy landscapes (FEL)

Applying PCA to analyze the FELs allows for a comprehensive understanding of the conformational dynamics of the system [36]. The analysis revealed that aloe-emodin binding to TRPV1 resulted in a single local minimum, in contrast to chrysophanol and capsazepine which exhibited three local minima, and emodin, which showed two local minima (Fig. 3b). Furthermore, physcion binding to TRPV1 displayed two local minima but occupied a substantial conformational space compared to the other compounds. Rhein binding to TRPV1 produced three local minima. These findings suggest that TRPV1 can readily access local minima when interacting with physcion.

#### Protein-ligand interaction

The interactions between each residue of TRPV1 and the ligand were calculated during the dynamic simulation. Figure 3c illustrates the temporal interaction profile between each residue of TRPV1 and the ligand. Normalization was conducted throughout the simulation period, as depicted in Fig. 3d, where a probability of 1 signifies that the TRPV1 residue interacted with the ligand over the 100 ns simulation, and a probability of 0.9 indicates that the ligand interacted with the residue for 90 ns during the simulation. The top 5 TRPV1 residues binding with the ligand were selected and displayed in Fig. 3d.

The analysis revealed that capsazepine specifically interacted with residues TYR511, THR550, SER512, LEU557, and ILE570, with probabilities of 0.925, 0.92, 0.83, 0.785, and 0.68. Aloe-emodin interacted with key residues essential for TRPV1 inhibition, namely THR550, ARG557, and GLU570. However, only the probability of THR550 exceeded 0.705, while the probabilities for ARG557 and GLU570 were 0.435 and 0.420, respectively. In contrast, chrysophanol bound to TRPV1 and interacted solely with the key residue GLU570, with a probability of 0.925. Emodin exhibited interactions with key TRPV1 residues, including LEU515, TYR511, and THR550, with probabilities of 0.800, 0.610, and 0.520, respectively. Physcion interacted with crucial residues such as ARG557, THR550, and TYR511, with probabilities of 0.950, 0.645, and 0.580. Moreover, rhein showed interactions with LEU515, THR550, and TYR511, with probabilities of 0.655, 0.650, and 0.650, respectively.

To rigorously validate the accuracy of our interaction detection methodology used in Fig. 3c, we performed the molecular docking and molecular dynamics (MD) simulation between capsaicin and TRPV1 using the same virtual screening pipeline. We then compared the resulting ligand-protein interactions against the well-characterized binding mode of capsaicin documented in the literature. In our study, the molecular docking successfully reproduced the known “tail-up, head-down” conformation of capsaicin within the vanilloid-binding pocket of TRPV1 (Fig. S2a). The top interacting residues identified in our MD simulations (ranked by contact frequency) were: Y513, E572, L517, T552, and Y555 (Fig. S2b). This aligns closely with prior studies reporting Y513, T552, L517, A568, and E572 as dominant residues (based on interaction fraction, defined as the percentage of frames exhibiting specific interactions) [37]. Notably, 4 out of the top 5 residues overlap between our work and published data. Furthermore, numerous additional residues known to interact with capsaicin (e.g., A667, F545, F593, I663, L664, R559, N553) were consistently detected by our method, corroborating the reference data (Fig. S2c-d) [37]. This high degree of correspondence between our MD-derived interaction profile for capsaicin and the established literature data strongly validates the accuracy and reliability of our interaction detection pipeline employed for analyzing ligand binding modes in the study.

Overall, emodin, physcion, and rhein demonstrated interactions with a greater number of key residues involved in TRPV1 inhibition compared to the other compounds, suggesting that these three compounds could establish strong interactions with TRPV1 and possess higher potential for TRPV1 inhibition.

#### MMPBSA analysis

The MM-PBSA methodology was employed to compute the binding free energy of the five compounds associated with TRPV1. The outcomes revealed that physcion exhibited the lowest binding free energy, with emodin ranking second. Chrysophanol displayed the highest binding free energy, followed by aloe-emodin and rhein. These findings suggested that physcion demonstrated the strongest affinity for TRPV1 compared to the other compounds (Table 1).

**Table 1:** Energetic components of the binding energy for five compounds in complex with TRPV1 using MM-PBSA (kJ/mol)

Components	Aloe-emodin	Chrysophanol	Emodin	Physcion	Rhein
van der Waal energy (ΔE_vdW_)	-191.471	-174.814	-205.730	-211.798	-152.133
Electrostatic energy (ΔE_elec_)	-51.749	-27.261	-57.229	-43.390	-20.567
^a^ ΔE_MM_	-243.221	-202.075	-262.959	-255.188	-172.700
Polar solvation energy (ΔG_pol_)	158.858	143.403	168.568	161.595	105.359
Non-polar solvation energy (ΔG_np_)	-29.289	-27.327	-31.974	-31.377	-23.278
Configurational entropy (-TΔS)	61.371	41.094	56.096	34.105	34.383
Binding energy (^b^ ΔG_Bind_)	-52.271	-44.904	-70.268	-90.864	-56.273

Note: ^a^ ΔE_MM_ = ΔE_vdW_ + ΔE_elec_, ^b^ ΔG_Bind_ = ΔE_MM_ + ΔG_pol_ + ΔG_np_ − TΔS

#### Calcium imaging

**Fig. 4:**
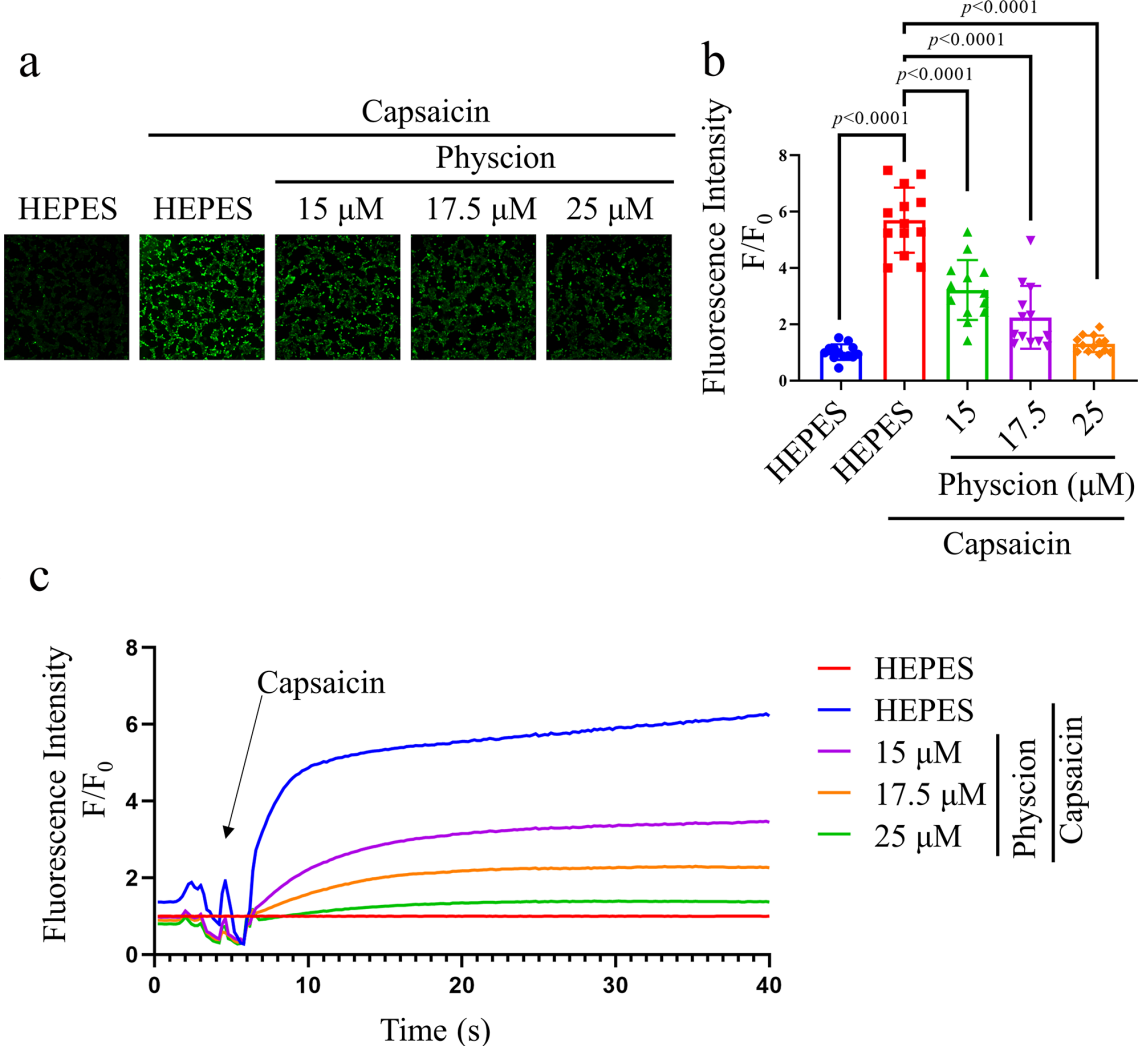
Calcium imaging depicting various concentrations (15–25 µM) of physcion on HEK293-hTRPV1 cells. (**a**) Illustrative images displaying the intracellular calcium fluorescence intensity of HEK293-hTRPV1 cells. (**b**) Normalized the average of relative calcium fluorescence intensity of physcion in HEK293-hTRPV1 cells. (**c**) Exemplary time-dependent profile of calcium fluorescence intensity in response to physcion in HEK293-hTRPV1 cells. Data are expressed as mean ± SD, Data were standardized to the HEPES control group. Statistical analysis by one-way ANOVA with Bonferroni’s post hoc test

Calcium imaging was conducted utilizing HEK293-hTRPV1 cellular models (Fig. 4). The application of capsaicin elicited a notable influx of calcium ions. The fluorescence intensity of HEPES was set as the baseline. Various concentrations of physcion, within the range of 15 to 25 µM, attenuated the calcium fluorescence triggered by capsaicin. These findings suggested that physcion exerted inhibitory effects on the calcium influx mediated by TRPV1.

### Pain behavioral tests

#### Acetic acid-induced abdominal stretching

**Fig. 5:**
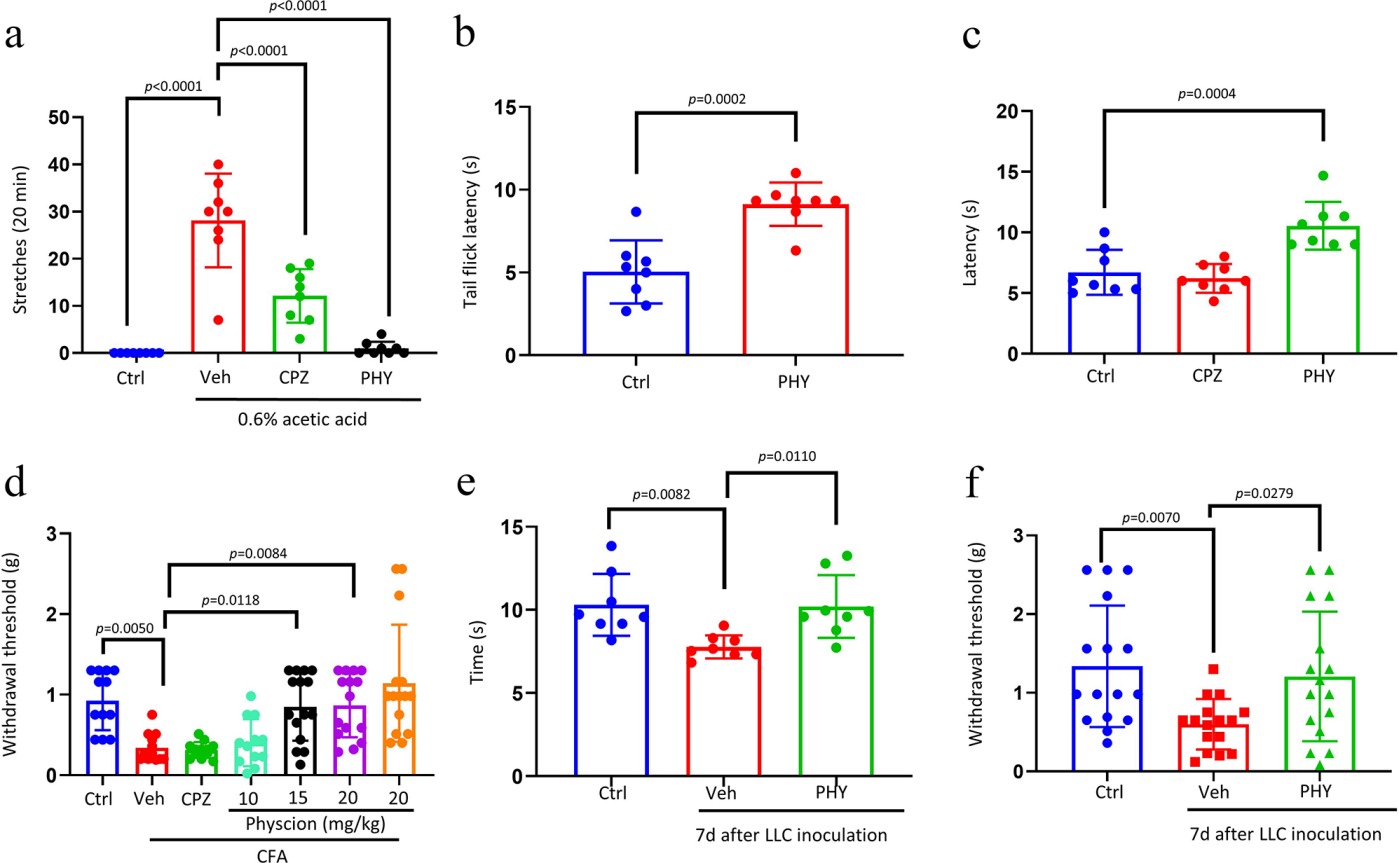
Assessment of the analgesic efficacy of Physcion across a variety of animal models. (**a**) The quantification of writhing episodes within 20 min following the administration of 0.6% acetic acid (*n* = 8). Thermal sensitivity was evaluated using the hot water tail-flick test (**b**) (*n* = 8) and hot plate assay (**c**) (*n* = 8). (**d**) The mechanical withdrawal threshold was determined through the von Frey test in the CFA-induced inflammatory pain model (*n* = 12–15). Thermal and mechanical sensitivities in the context of bone cancer pain induced by LLC inoculation were assessed viathe hot plate test (**e**) (*n* = 8) and von Frey test (**f**) (*n* = 16). The dose of CPZ was 20 mg/kg. The data are presented as mean ± standard deviation. Statistical analyses were performed using unpaired two-tailed Student’s t-test for (**b**) and one-way ANOVA with Bonferroni’s post hoc test for (**a**), (**c**), (**d**), (**e**), and (**f**). Ctrl indicates the control group; Veh indicates the vehicle group; CPZ indicates the capsazepine group; CFA indicates complete Freund’s adjuvant; LLC indicates Lewis lung carcinoma

Following the administration of 0.6% acetic acid, trunk curling and limb extension were observed in mice, indicative of stretching response (Fig. 5a). Upon administering 20 mg/kg CPZ, the number of stretches significantly decreased compared to the control group. Furthermore, administering 20 mg/kg physcion resulted in a more pronounced reduction in stretch number compared to the CPZ group.

#### Hot water tail-flick test

The hot-water tail-flick test was performed to assess heat pain sensitivity in mice. Treatment with 20 mg/kg of physcion elevated the thermal threshold in mice compared to the control group (Fig. 5b).

#### Hot plate test

In the hot plate test, the administration of 20 mg/kg CPZ did not prolong the latency of the hot plate test. Conversely, treatment with 20 mg/kg of physcion significantly elevated the thermal threshold in mice compared to the control group (Fig. 5c).

#### CFA-induced inflammatory pain

The injection of CFA induced mechanical allodynia and thermal hyperalgesia, which were assessed using the von Frey test to measure the paw withdrawal threshold [38]. The findings revealed a significant decrease in the paw withdrawal threshold following CFA injection. Treatment with 20 mg/kg of CPZ did not restore the paw withdrawal threshold (Fig. 5d). However, the administration of 15 or 20 mg/kg of physcion significantly increased the withdrawal threshold. Furthermore, intraperitoneal administration of 20 mg/kg of physcion without CFA treatment did not affect the paw withdrawal threshold.

#### Bone cancer pain

The intra-femoral inoculation of Lewis lung carcinoma (LLC) cells is a well-established model for studying bone cancer pain and evaluating the analgesic properties of pharmaceutical agents [39]. The hot plate and von Frey tests assessed the physcion’s thermal and mechanical analgesic effects (Fig. 5e-f). Results indicated that LLC inoculation significantly reduced the latency in the hot plate test and the paw withdrawal threshold in the von Frey test. However, treatment with 20 mg/kg of physcion notably increased both the latency in the hot plate test and the paw withdrawal threshold in the von Frey test.

### The effect of physcion on inflammatory mediators in plasma and DRG during CFA-induced inflammatory pain in mice

**Fig. 6:**
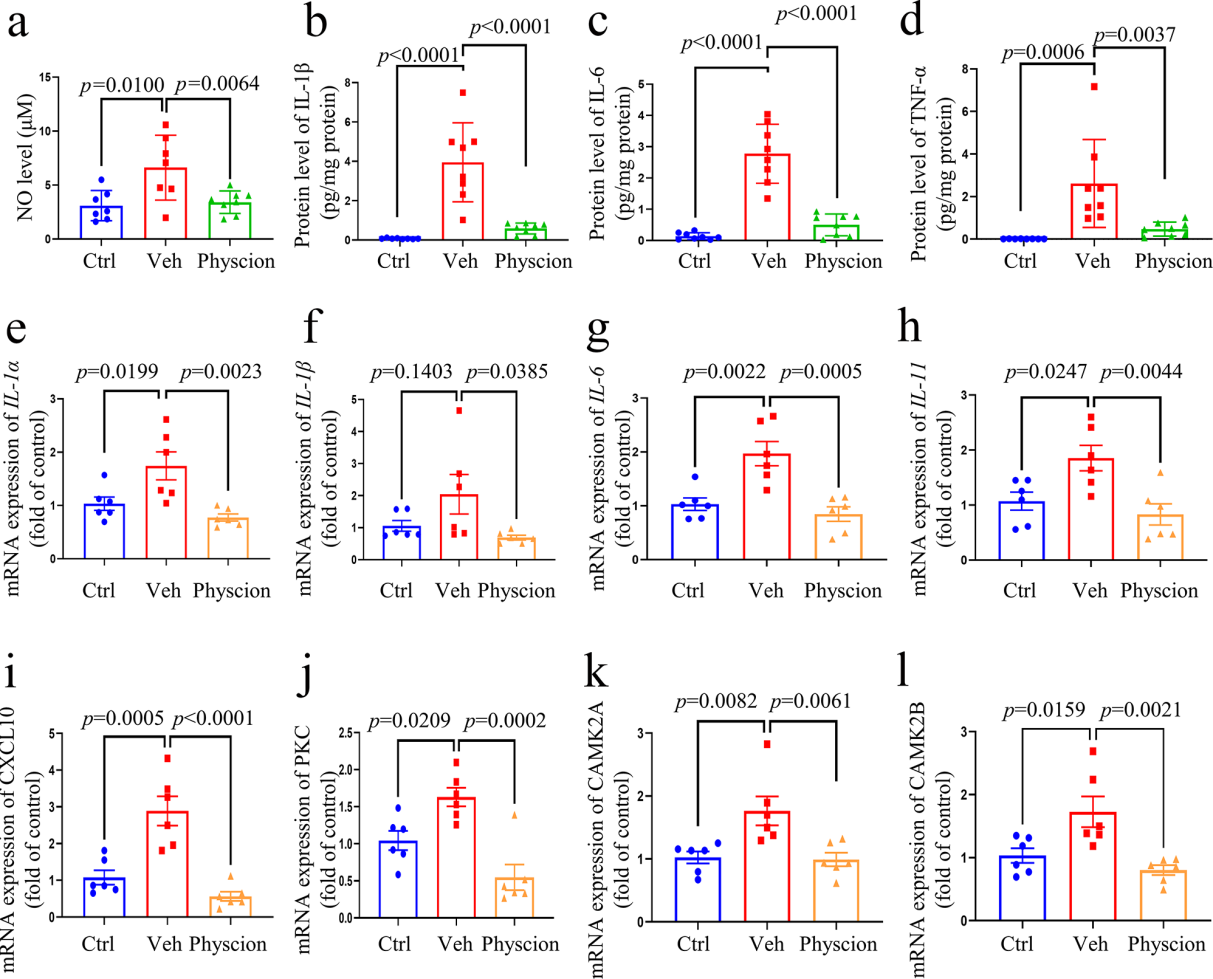
Impact of physcion on CFA-induced inflammatory pain in the plasma and the DRG of mice. (**a**-**d**) Plasma analysis: Influence of physcion on the secretion levels of NO, IL-1β, IL-6, and TNF-α (*n* = 7–8); (**e**-**l**) mRNA analysis of DRG: Influence of physcion on the mRNA expression levels of *IL-1α*, *IL-1β*, *IL-6*, *IL-11*, *CXCL10*, *PKC*, *CAMK2A*, and *CAMK2B* (*n* = 6). Data are presented as mean ± SD, statistical analysis by one-way ANOVA with Bonferroni’s post hoc test. Ctrl indicates the control group; Veh indicates the vehicle group

To explore the underlying mechanism of physcion’s analgesic effect, we examined the inflammatory cytokines provoked by CFA-induced inflammatory pain in mice’s plasma and DRG. Our findings revealed that CFA administration led to an elevation in the secretion of inflammatory cytokines such as NO, IL-1β, IL-6, and TNF-α (Fig. 6a-d). Interestingly, treatment with 20 mg/kg of physcion notably attenuated the levels of these inflammatory cytokines.

Moreover, concerning mRNA expression, the injection of CFA not only impacted the mRNA levels of inflammatory genes like *IL-1α*, *IL-1β*, *IL-6*, *IL-11*, and *CXCL10* but also influenced the expression of calcium-related genes such as *PKC*, *CAMK2A*, and *CAMK2B* (Fig. 6e-l). Conversely, treatment with 20 mg/kg of physcion markedly reduced the mRNA expression of both inflammatory and calcium-related genes.

### The anti-inflammatory effect of physcion on TNF-α-induced RAW264.7 cells

**Fig. 7:**
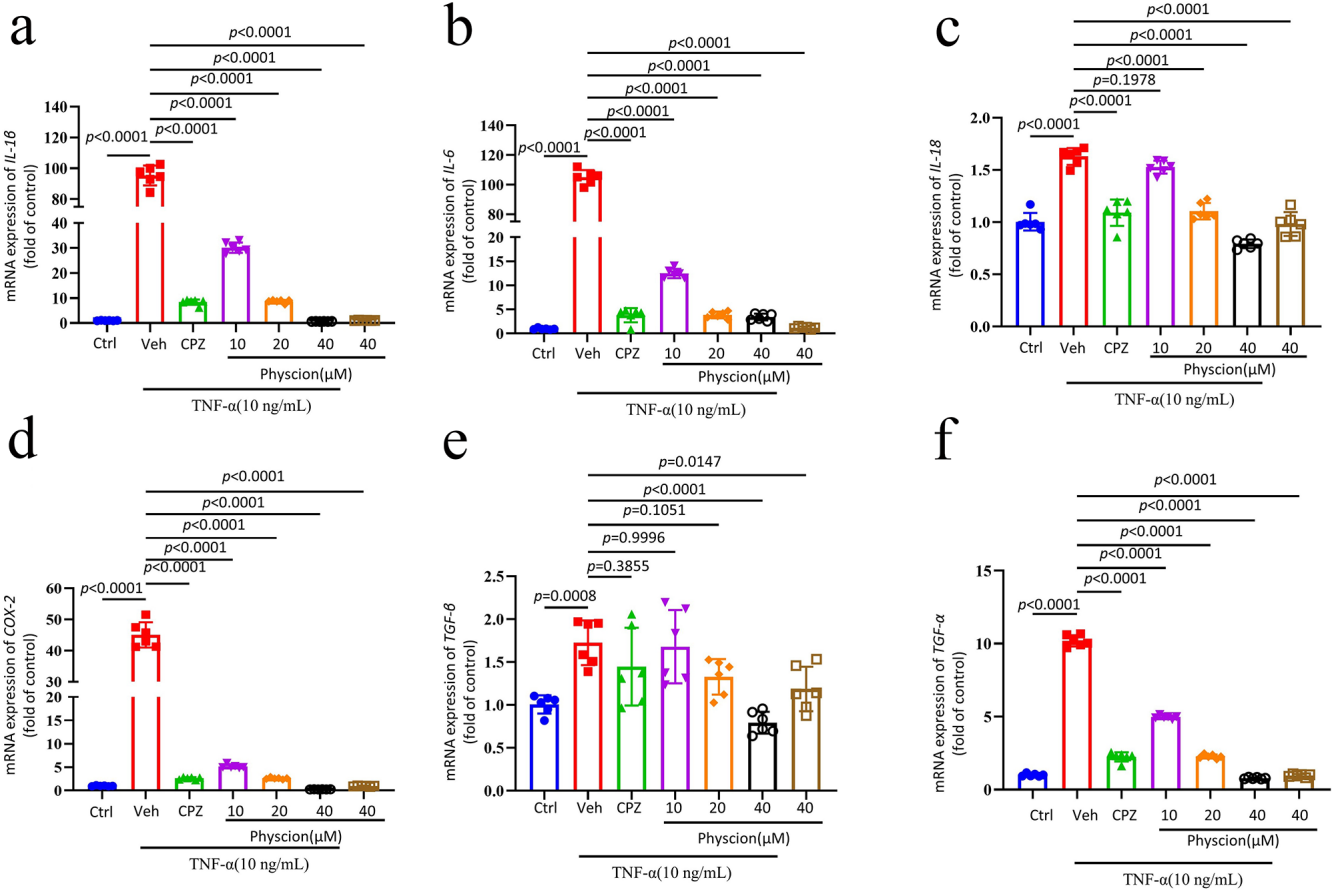
(**a**-**f**) The impact of physcion on the mRNA expression of *IL-1β*, *IL-6*, *IL-18*, *COX-2*, *TGF-β*, and *TNF-α* in TNF-α-stimulated RAW264.7 cells (*n* = 6). The dose of CPZ was 20 µM. Results are expressed as mean ± SD. Statistical evaluation was conducted using one-way ANOVA followed by Bonferroni’s post hoc analysis. In the experimental context, Ctrl denotes the control group, Veh represents the vehicle group, and CPZ signifies the capsazepine group

Given the pivotal involvement of TNF-α in inflammation progression and our prior findings indicating the ability of CFA to induce TNF-α synthesis and subsequently trigger inflammation, our study concentrated on investigating the anti-inflammatory properties of physcion on TNF-α-stimulated RAW264.7 cells. Stimulation with 10 ng/ml of TNF-α resulted in a significant upregulation of mRNA expression of *IL-1β*, *IL-6*, *IL-18*, *COX-2*, *TGF-β*, and *TNF-α* (Fig. 7a-f). Treatment with 40 µM of CPZ decreased the mRNA expression levels, whereas varying concentrations of physcion ranging from 10 to 40 µM markedly attenuated the expression of these inflammatory genes.

### NF-κB/MAPK signaling pathways mediated the anti-inflammatory effect of physcion

**Fig. 8:**
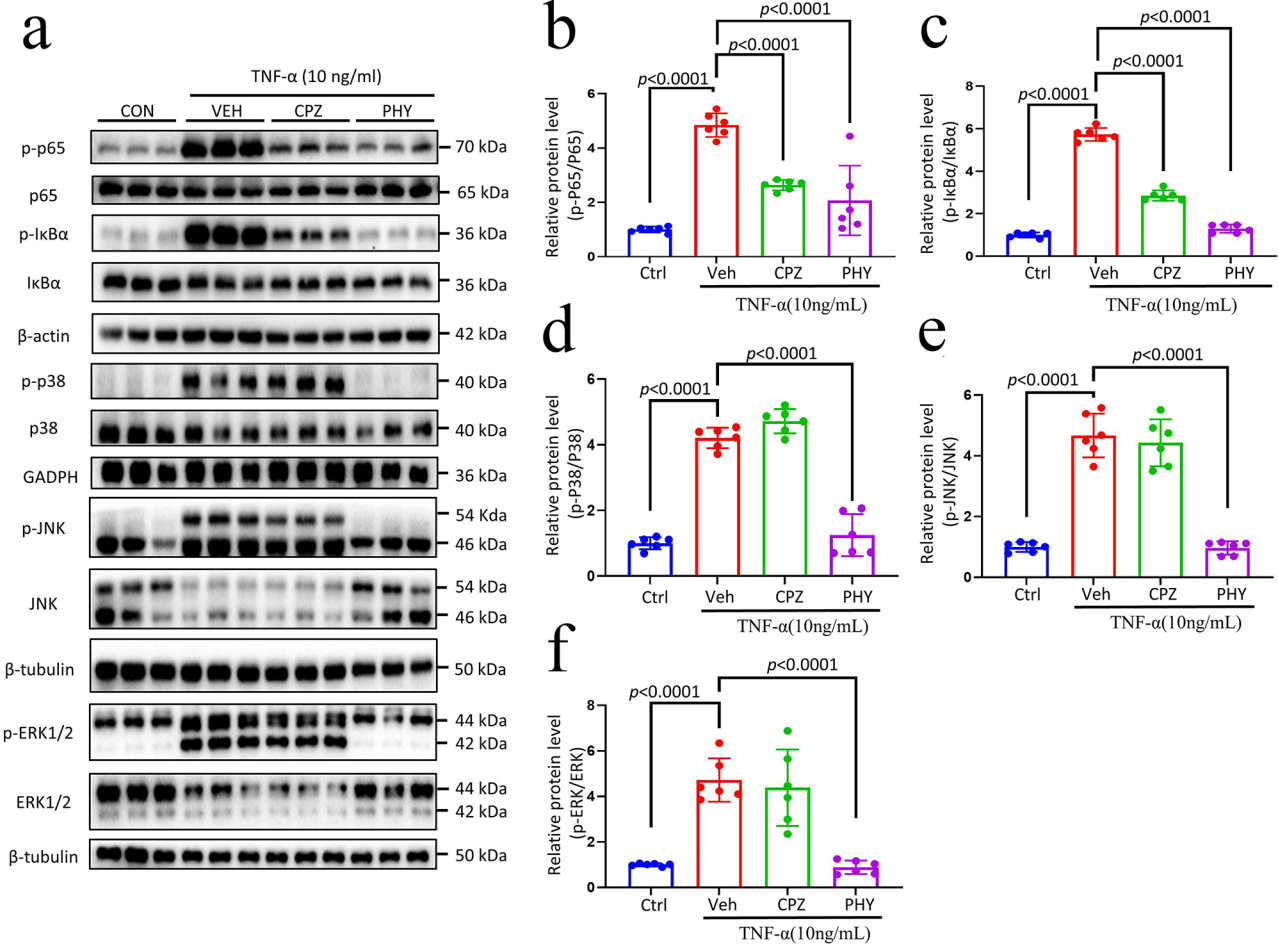
Physcion’s impact on NF-κB and MAPK signaling pathways. RAW264.7 cells underwent treatment with CPZ (40 µM) or physcion (40 µM) for 1 h. Subsequently, (**a**) whole-cell lysates were obtained and subjected to immunoblotting using antibodies specific to p-p65, p65, p-IκBα, IκBα, p-p38, p38, p-JNK, JNK, p-ERK1/2, ERK1/2, β-actin, GAPDH, and β-tubulin. The fold change was assessed post-normalization with β-actin, GAPDH, or β-tubulin. The accompanying right panels depict the fold change as compared to the control: p-p65/p65 (**b**), p-IκBα/IκBα (**c**), p-p38/p38 (**d**), p-JNK/JNK (**e**), p-ERK1/2/ERK1/2 (**f**) (*n* = 6). The data is represented as mean ± SD. Statistical analysis was performed using one-way ANOVA with Bonferroni’s post hoc test. In the experimental context, Ctrl denotes the control group; Veh represents the vehicle group; CPZ signifies the capsazepine group; PHY indicates the physcion group

The NF-κB/MAPK signaling cascade is triggered upon exposure to TNF-α [40]. Previous studies have demonstrated that blocking NF-κB can mitigate mechanically and thermally induced pain in rats caused by CFA [41]. This study aimed to investigate the anti-inflammatory properties of physcion on the NF-κB and MAPK signaling pathways. The findings revealed that exposure to 10 ng/ml TNF-α resulted in the increased phosphorylation of p65, IκBα, p38, JNK, and ERK1/2 (Fig. 8). Furthermore, treatment with 40 µM of CPZ attenuated the phosphorylation of p65 and IκBα. At the same time, it did not affect the phosphorylation of p38, JNK, and ERK1/2. Conversely, treatment with 40 µM of physcion exhibited a significant inhibitory impact on the phosphorylation of p65, IκBα, p38, JNK, and ERK1/2. These results suggested that physcion’s anti-inflammatory property was attributed to its ability to suppress the NF-κB/MAPK signaling pathways.

## Discussion

This study aims to discover a novel painkiller from natural products through virtual screening. Our investigation revealed that five compounds exhibited significant affinity towards TRPV1. Subsequent analysis identified physcion as the most potent binder to TRPV1 through molecular dynamics simulations. Calcium imaging experiments demonstrated physcion’s ability to inhibit calcium influx through TRPV1. Furthermore, physcion demonstrated pronounced analgesic effects in various animal models, including acetic acid-induced, thermal-induced, CFA-induced inflammatory pain, and LLC cell-induced bone cancer pain. Additionally, physcion exhibited notable anti-inflammatory properties by reducing inflammatory cytokine secretion or mRNA expression in CFA-induced inflammatory pain models. These effects may be attributed to suppressing the NF-κB and MAPK signaling pathways. Our findings suggest that physcion holds promise as a potential candidate for developing novel analgesic agents.

TRPV1 plays a crucial role in pain transmission and modulation, responding to various stimuli such as capsaicin, heat, and pro-inflammatory compounds [6]. The docking result showed that all the major rhubarb components could interact with the key residues for inhibition of TRPV1. However, aloe-emodin and chrysophanol could interact with TRPV1 on only one key residue, while the other three compounds could interact with TRPV1 more than three key residues. The result demonstrated that aloe-emodin and chrysophanol show less potency in the inhibition of TRPV1.

The molecular docking results may not entirely reflect the interactions between TRPV1 and the five components under physiological conditions. Molecular dynamics simulations were conducted to assess the stability of the interactions between TRPV1 and the compounds. Regarding the RMSD of the protein or ligand, the findings indicated that physcion, when bound to TRPV1, resulted in a more stable structure. The reduced Rg value and relatively lower SASA values further supported the notion that physcion bound to TRPV1, forming a more compact structure. Additionally, the hydrogen bond analysis underscored the binding affinity between TRPV1 and physcion. Moreover, the PCA results illustrated that physcion binding to TRPV1 occupied a smaller spatial volume, while the FEL analysis indicated the formation of a local minimum upon their combination. These findings suggested that TRPV1 could readily achieve the lowest energy state and attain a highly stable structure following interaction with physcion. Additionally, the analysis of contact numbers between TRPV1 and the compounds during MD simulations revealed that emodin, physcion, and rhein established numerous interactions with key residues of TRPV1. Regarding the binding free energy, physcion exhibited the highest affinity upon binding with TRPV1. Integrating the insights from molecular docking and MD simulations, physcion emerged as the most potent inhibitor of TRPV1. Physcion exhibited comparable RMSD values to TRPV1 and lower RMSD values for the ligand when compared to capsazepine. Moreover, during MD simulation, Physcion demonstrated analogous higher Rg values, lower hydrogen bond counts, and lower SASA values than capsazepine. Importantly, they also shared similar interactive residues, specifically LEU557, THR550, and TYR511. These findings suggested that physcion may elicit a similarly inhibitory effect on TRPV1, akin to capsazepine.

Regarding the gating mechanism of TRPV1, in a previous investigation by Yang et al., it was elucidated that upon capsaicin binding to TRPV1, an allosteric conformational shift is initiated in the ligand-binding site, which triggers a conformational wave propagating from the ligand-binding pocket towards the S6 bundle, traversing through the S4-S5 linker, and culminating in the selectivity filter region [42]. Furthermore, the study by Yin et al. demonstrated that the TRPV1 agonist 6-shogaol engages with the TRPV1 channel via hydrogen bonds formed between the neck and T551, as well as the head and E571 [43]. Additionally, the capsazepine-bound structure, determined in either amphipol or nanodisc environments, revealed that this competitive vanilloid antagonist occupies the same hydrophobic pocket as RTX, albeit without facilitating the critical interaction between Arg557 and Glu570, a scenario similar to our simulation outcomes as discussed in the TRPV1 structures [34]. Concurrently, analysis of another TRPV1 inhibitor, SB-366,791, uncovered that SB-366,791 also engages in hydrophobic interactions with L515 in S3, as well as L547, T550, and L553 in S4 [33]. Both agonists (capsaicin and resiniferatoxin) as well as competitive antagonists (capsazepine and SB-366791) have the ability to bind to this particular site on the TRPV1 receptor. The agonists facilitate the formation of a salt bridge between Arg557 and Glu570, causing S5 to be drawn towards the S1-S4 domain, thereby resulting in the opening of the channel. Conversely, the antagonists discourage the interaction between Arg557 and Glu570, thereby promoting the stabilization of the closed state [44]. Furthermore, physcion demonstrated analogous attributes to capsazepine in MD simulation, such as RMSD, Rg, hydrogen bonding, and SASA. Notably, Physcion demonstrates robust binding affinity towards T550 and L553, suggesting a partial similarity in the modulation of TRPV1 gating by physcion akin to the inhibitory effects observed with TRPV1 inhibitors capsazepine or SB-36,679.

While utilizing MD simulations to predict potential binding poses and allosteric effects of capsazepine or physicion, trajectory analysis reveals some common characteristics upon binding of physcion or capsazepine to TRPV1, such as relatively stable RMSD, smaller Rg and SASA values, increased hydrogen bonds, and similarity in the amino acids engaged during the MD simulation. However, disparities are noted in PCA analysis and the free energy landscape. Therefore, further elucidation of the functional and structural changes induced by physcion or capsazepine on TRPV1 will necessitate complementary approaches, such as patch-clamp electrophysiology and cryo-EM structural resolution.

The previous study showed that emodin proficiently impedes the up-regulation of TRPV1 and pro-inflammatory cytokines [45]. Nonetheless, the study on emodin did not assess its functional impact through methods such as calcium imaging or patch clamp to validate the inhibition of TRPV1. Calcium imaging also corroborated the inhibitory effect of physcion in suppressing the calcium influx through TRPV1. Consequently, subsequent in vivo studies were conducted to delve into the analgesic potential of TRPV1.

Physcion has been demonstrated to possess various effects, including anticancer, antimicrobial, and antioxidant properties [46]; given the notable inhibitory effect on the calcium influx of TRPV1 observed with physcion and the unexplored analgesic potential, the subsequent study aimed to investigate the analgesic properties of physcion utilizing diverse animal models. The findings revealed that physcion attenuated the writhing response induced by acetic acid, prolonged the latency in the hot water tail-flick and hot plate assays, and lowered the withdrawal threshold in pain models induced by complete Freund’s adjuvant (CFA) and bone cancer. These results suggested that physcion elevated the mechanical and thermal pain thresholds across acute, inflammatory, chronic, and bone cancer pain conditions.

Nevertheless, the TRPV1 inhibitor CPZ did not exhibit a substantial impact on thermal or mechanical pain. This observation aligns with findings reported by other researchers [47–49]. CPZ can not only suppress the activation of TRPV1 but also inhibit the activation of TRPA1. In terms of thermal pain, CPZ was found to be ineffective in providing antinociception in various rat pain models, including the tail-flick, hot-plate, and carrageenan-induced inflammatory mechanical hyperalgesia paradigms [50]. In terms of mechanical pain, tactile allodynia is known to be mediated by Aβ-afferent fibers [51]. This could explain the observed inefficacy of capsazepine in the von Frey test, as it does not affect Aβ-fibers. Moreover, Walker et al. documented species-specific differences in capsazepine’s antiallodynic effects across guinea pigs, mice, and rats, showing its ineffectiveness in alleviating chronic pain symptoms and mechanical hyperalgesia in mice and rats compared to its efficacy in guinea pigs [49]. According to a previous study, the intraperitoneal administration of capsazepine was shown to decrease heat hyperalgesia but had no impact on mechanical and cold allodynia in a murine model of cancer-related pain [52]. This result aligns with our own observations. However, the intricate mechanisms still remain to be elucidated.

The study examined the inflammatory cytokines and mRNA expression to elucidate the analgesic mechanism of physcion. NO, IL-1α, IL-1β, IL-6, and TNF-α are pivotal signaling cytokines that are instrumental in the pathogenesis of inflammation [53]. IL-11 functions as a pro-inflammatory protein, while CXCL10 is recognized for facilitating leukocyte trafficking to inflamed tissues; heightened levels of CXCL10 may intensify the inflammatory response [54]. Multiple phosphorylation sites for protein kinase C (PKC) are present on TRPV1, influencing channel sensitization and desensitization [55]. Calcium influx triggers the activation of CaMK2A and CaMK2B, resulting in subsequent calcium-calmodulin (Ca^2+^-CaM) binding [56]. Our results suggested that physcion inhibited the secretion and mRNA expression of inflammatory cytokines and modulated the mRNA levels of calcium-related genes. These findings highlighted that physcion exerted analgesic properties by regulating TRPV1, consequently attenuating inflammation.

RAW264.7 cells were utilized in this investigation to elucidate the anti-inflammatory mechanism of physcion. The findings demonstrated that physcion mitigated the mRNA expression of inflammatory genes. Furthermore, the impact of physcion on the NF-κB and MAPK signaling pathways was also examined. TNF-α can activate the phosphorylation of IκBα, p65, p38, JNK, and ERK1/2 [57, 58]. Physcion effectively hindered the phosphorylation of p65, IκBα, p38, JNK, and ERK1/2 induced by TNF-α. These results suggested that physcion inhibited the calcium influx via TRPV1, modulated the NF-κB and MAPK signaling pathways, suppressed inflammatory cytokines, and exerted a notable analgesic effect.

Activation of TRPV1, a non-selective cation channel, triggers significant Ca²⁺ influx. Elevated intracellular Ca²⁺ activates calmodulin (CaM), subsequently stimulating calmodulin-dependent kinase II (CaMKII) and protein kinase C (PKC). Importantly, CaMKII can phosphorylate the IKKβ subunit of the IKK complex, potentially leading to IκBα degradation, NF-κB nuclear translocation, and transcription of inflammatory genes like IL-1β and TNF-α [59]. However, our data (Figure. 8) indicated a mechanistic caution: the selective TRPV1 antagonist CPZ significantly blocked channel activity but showed a comparatively weaker effect on the NF-κB pathway and downstream inflammatory cytokines. Conversely, physcion demonstrated significantly greater suppression of NF-κB activation and reduced IL-1β/TNF-α mRNA/protein levels both in vitro and in vivo, compared to CPZ. This key observation may reflect that physcion’s potent anti-inflammatory effects likely involved additional mechanisms independent or downstream of TRPV1 blockade.

Previous research has demonstrated that capsazepine inhibited the NF-κB signaling pathway in LPS-stimulated RAW264.7 macrophages [60]. While the anti-inflammatory effects of capsazepine have historically been attributed to TRPV1 inhibition, recent studies suggested an alternative or additional mechanism involving the targeting of Syntaxin 7 (Stx7). Stx7 is recognized as a protein involved in inflammasome activation. Furthermore, previous study indicated that capsazepine exerted its anti-inflammatory effects more specifically by inhibiting astrocyte activation rather than microglia [61]. For comparison, physcion has been shown to inhibit TNF-α-induced inflammation in RAW264.7 macrophages. However, studies on physcion have not yet directly compared its effects under LPS stimulation or across different cell types like microglia or astrocytes. Notably, physcion demonstrated superior efficacy compared to capsazepine in our study. This enhanced effect may stem from physcion targeting pathways beyond TRPV1.

The divergence in TRPV1 species sensitivity is noteworthy, with rodent and human TRPV1 channels displaying heightened responsiveness to vanilloids, showcasing EC_50_ values at the nanomolar level. In contrast, the TRPV1 channels of frog (*Xenopus tropicalis*) and rabbit (*Oryctolagus cuniculus*) exhibit a reduced sensitivity to capsaicin [62]. Virtual screening in this study utilized rodent-derived TRPV1. The amino acid sequence homology between human and rodent TRPV1 channels is approximately 80-90%; however, variations at critical sites significantly impact functionality. Capsazepine functions as a competitive antagonist of TRPV1. Previous studies have unequivocally demonstrated the competitive antagonistic effects of this molecule on both rTRPV1 and hTRPV1 at 22 °C and 37 °C [63]. The potency of capsazepine on TRPV1 remains unaffected by temperature [64], yet compared to rTRPV1, its efficacy on hTRPV1 is enhanced by approximately 6-fold. Consequently, additional research on human-derived TRPV1 is imperative before advancing physcion into clinical investigation.

## Conclusion

**Fig. 9:**
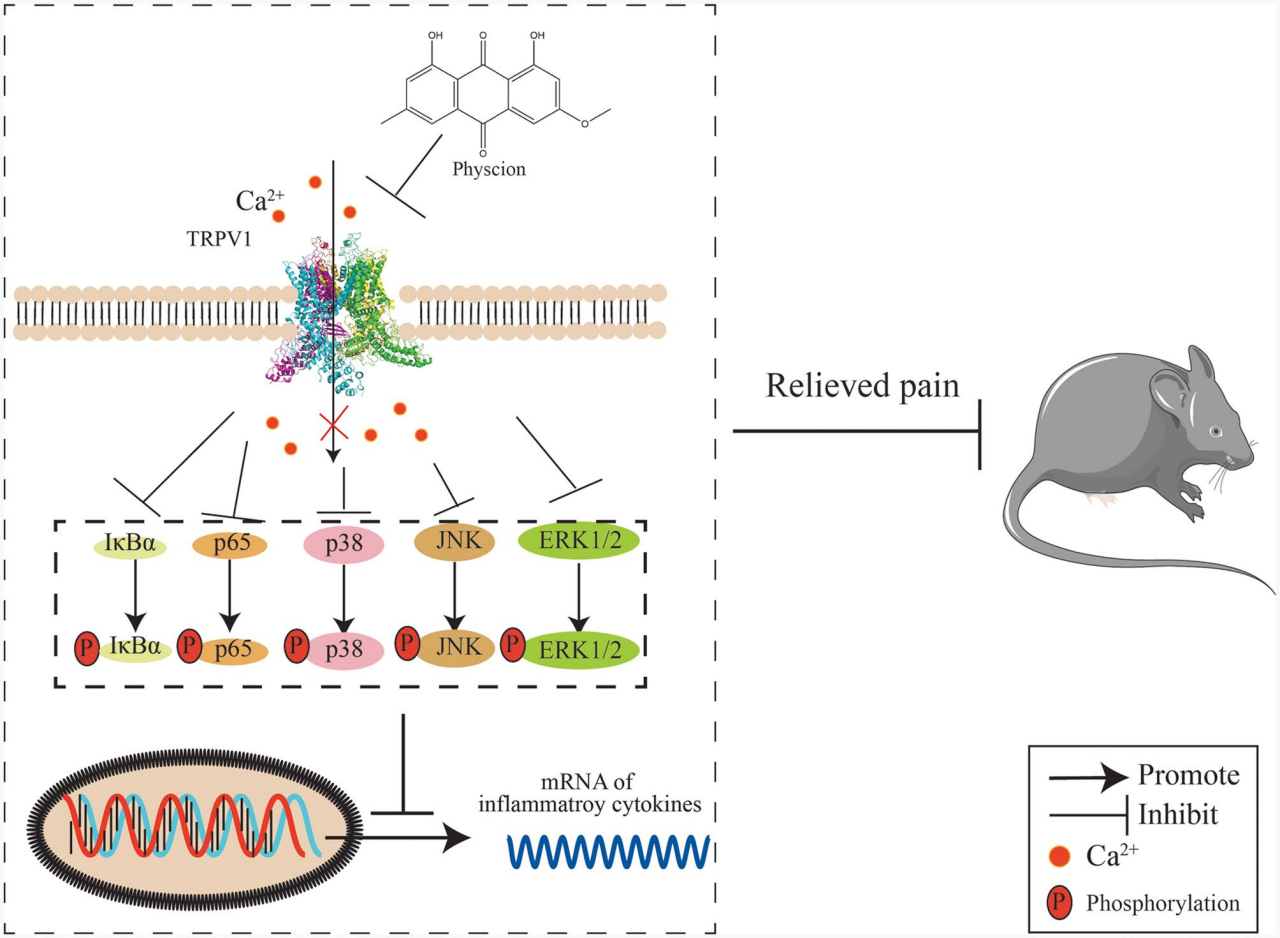
Proposed mechanism of physcion-mediated analgesia via TRPV1 signaling suppression. Physcion inhibits calcium influx through TRPV1 channels, thereby attenuating phosphorylation of the NF-κB (IκBα/p65) and MAPK (p38/JNK/ERK1/2) signaling pathways. This suppression downregulates mRNA expression of pro-inflammatory genes (e.g., TNF-α, IL-1β, IL-6), ultimately relieving pain behaviors in a mouse model of pain

In summary, our study utilized virtual screening to elucidate the potential inhibitor of TRPV1. The results revealed that these compounds achieved the highest docking scores when interacting with TRPV1. Molecular dynamics simulations indicated that physcion displayed the most excellent efficacy in inhibiting TRPV1, confirmed by calcium imaging. Furthermore, physcion demonstrated a significant capacity to alleviate chronic, thermal, acute, inflammatory, and bone cancer-related pain. Its mechanism of action may involve the suppression of TRPV1 and inhibition of the NF-κB and MAPK signaling pathways, thereby reducing inflammatory cytokine levels and consequently alleviating pain (Fig. 9). Even at 650 mg/kg [65], physcion did not induce mortality in rats. In addition, SwissADME prediction indicated that physcion was unlikely to cross the blood-brain barrier, suggesting its primary site of action is the peripheral nervous system. By acting peripherally, physcion has the potential to offer improved safety over opioid analgesics, as it may circumvent the serious central side effects of abuse liability, addiction, and life-threatening respiratory depression. At a dose of 20 mg/kg, physcion exhibited excellent analgesic effects and did not cause hypothermic effect (Figure. S3), indicating its potential as a promising analgesic agent.

## Electronic supplementary material

Below is the link to the electronic supplementary material.

Supplementary Material 1

## Data Availability

The original contributions presented in the study are included in the article Supplemental Table, and further inquiries can be directed to the corresponding author.

## Abbreviations

ATCC: American type culture collection
CFA: Complete freund’s adjuvant
CGRP: Calcitonin gene-related peptide
COX-2: Cyclooxygenase 2
CPZ: Capsazepine
DRG: Dorsal root ganglia
ELISA: Enzyme-linked immunosorbent assay
FBS: Fetal bovine serum
FEL: Free energy landscape
GAPDH: Glyceraldehyde 3-phosphate dehydrogenase
HEK293: Human embryonic kidney 293 cells
IL-1β: Interleukin-1 beta
IL-6: Interleukin-6
LLC: Lewis lung carcinoma
MAPK: Mitogen-activated protein kinases
MD: Molecular dynamics
NF-κB: Nuclear-factor kappa-light-chain enhancer of activated B cells
NO: Nitric oxide
NPT: Isothermal-isobaric ensemble
NVT: Canonical ensemble
PBS: Phosphate-buffered saline
PCA: Principal component analysis
PDB: Protein data bank
PLIP: Protein-ligand interaction profiler
PMSF: Protease inhibitor phenylmethanesulfonyl fluoride
PVDF: Polyvinylidene difluoride
Rg: Radius of gyration
RIPA: Radioimmunoprecipitation assay lysis buffer
RMSD: Root mean square deviation
SASA: Solvent-accessible surface area
SD: Standard deviation
SMILES: Simplified Molecular Input Line Entry System
SPF: Specific pathogen-free
TCM: Traditional Chinese medicine
TNF-α: Tumor necrosis factor-α
TRPV1: Transient receptor potential cation channel subfamily V member 1
