# Supplementary material for: Pharmacological evaluation of physcion as a TRPV1 inhibitor with multimodal analgesic efficacy in experimental pain models
Source: Biol Res. 2025 Jul 10;58:48. doi: 10.1186/s40659-025-00630-5 (PMC12243309; doi:10.1186/s40659-025-00630-5)
Supplement: Supplementary file 1 — Supplementary Material 1 [file 40659_2025_630_MOESM1_ESM.docx]

Supplementary material

Pharmacological Evaluation of Physcion as a TRPV1 Inhibitor with Multimodal Analgesic Efficacy in Experimental Pain Models

Hanbin Chen^a,#^, Guanghong Li^a,#^, Lin Deng^b^, Shuli Li^c^, Songqiang Huang^d^, Simon Ming-Yuen Lee^c,e,f,g,h,i,j^, Xiaowei Nie^k,*^, Jin-Song Bian^a,*^

^a^ Department of Pharmacology, Joint Laboratory of Guangdong-Hong Kong Universities for Vascular Homeostasis and Diseases, SUSTech Homeostatic Medicine Institute, School of Medicine, Southern University of Science and Technology, Shenzhen, China

^b^ Department of Cardiology, The Eighth Affiliated Hospital of Sun Yat-sen University, Shenzhen, Guangdong, China

^c^ State Key Laboratory of Quality Research in Chinese Medicine and Institute of Chinese Medical Sciences, University of Macau, Macao, China

^d^ Affiliated Hospital of Hunan University, School of Biomedical Sciences, Hunan University, Changsha, Hunan, China

^e^ Department of Food Science and Nutrition, The Hong Kong Polytechnic University, Hung Hom, Hong Kong, China

^f^ PolyU-BGI Joint Research Centre for Genomics and Synthetic Biology in Global Ocean Resources, The Hong Kong Polytechnic University, Hung Hom, Hong Kong, China

^g^ Research Centre for Chinese Medicine Innovation, The Hong Kong Polytechnic University, Hung Hom, Hong Kong, China

^h^ State Key Laboratory of Chemical Biology and Drug Discovery, The Hong Kong Polytechnic University, Hung Hom, HongKong, China

^i^ Research Institute for Future Food, The Hong Kong Polytechnic University, Hung Hom, Hong Kong, China

^j^ Research Institute for Smart Ageing, The Hong Kong Polytechnic University, Hung Hom, Hong Kong, China

^k^ Department of Human Cell Biology and Genetics, School of Medicine, Southern University of Science and Technology

^#^ H. Chen and G. Li contributed equally.

* Corresponding author: Xiaowei Nie (niexw@sustech.edu.cn) and Jin-Song Bian (bianjs@sustech.edu.cn)


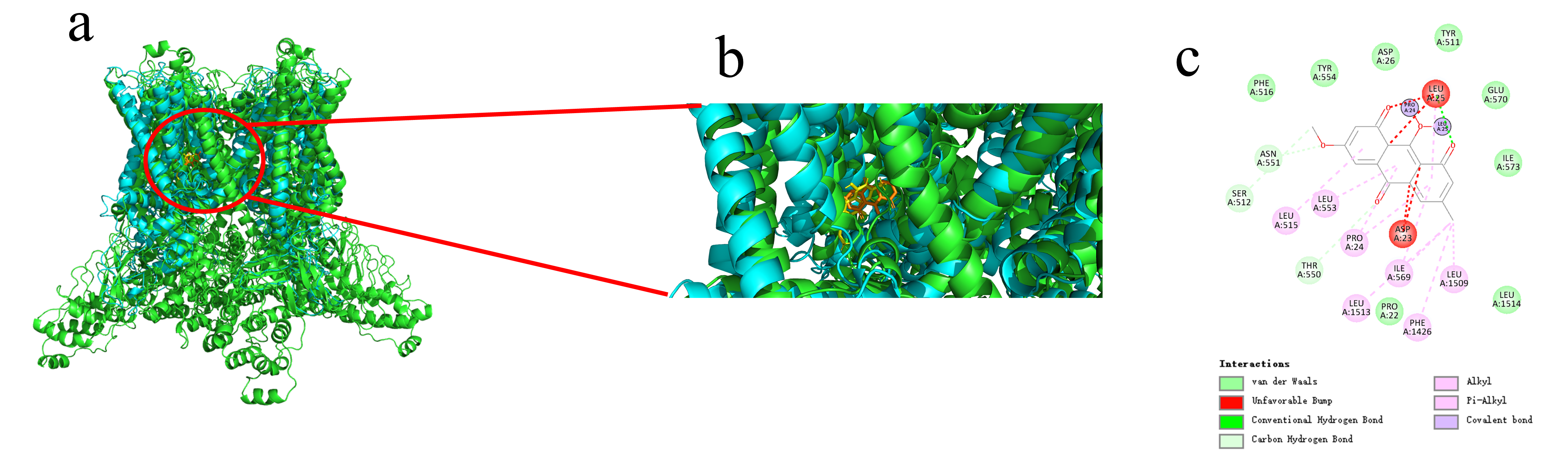
Figure S1. The docking configuration of physcion with TRPV1 generated by Vina-GPU and AlphaFold3. AlphaFold3 metrics: Aggregate score (0.6); Mean pLDDT (68); global PDE (1.9975); pTM (0.6709); ipTM (0.5823). (a) Comparative analysis of the docking configurations produced by Vina-GPU (cyan) and AlphaFold3 (green). (b) Localization of physcion as predicted by Vina-GPU (yellow) and AlphaFold3 (orange). (c) Visualization of the molecular interactions between TRPV1 and physcion predicted by AlphaFold3.


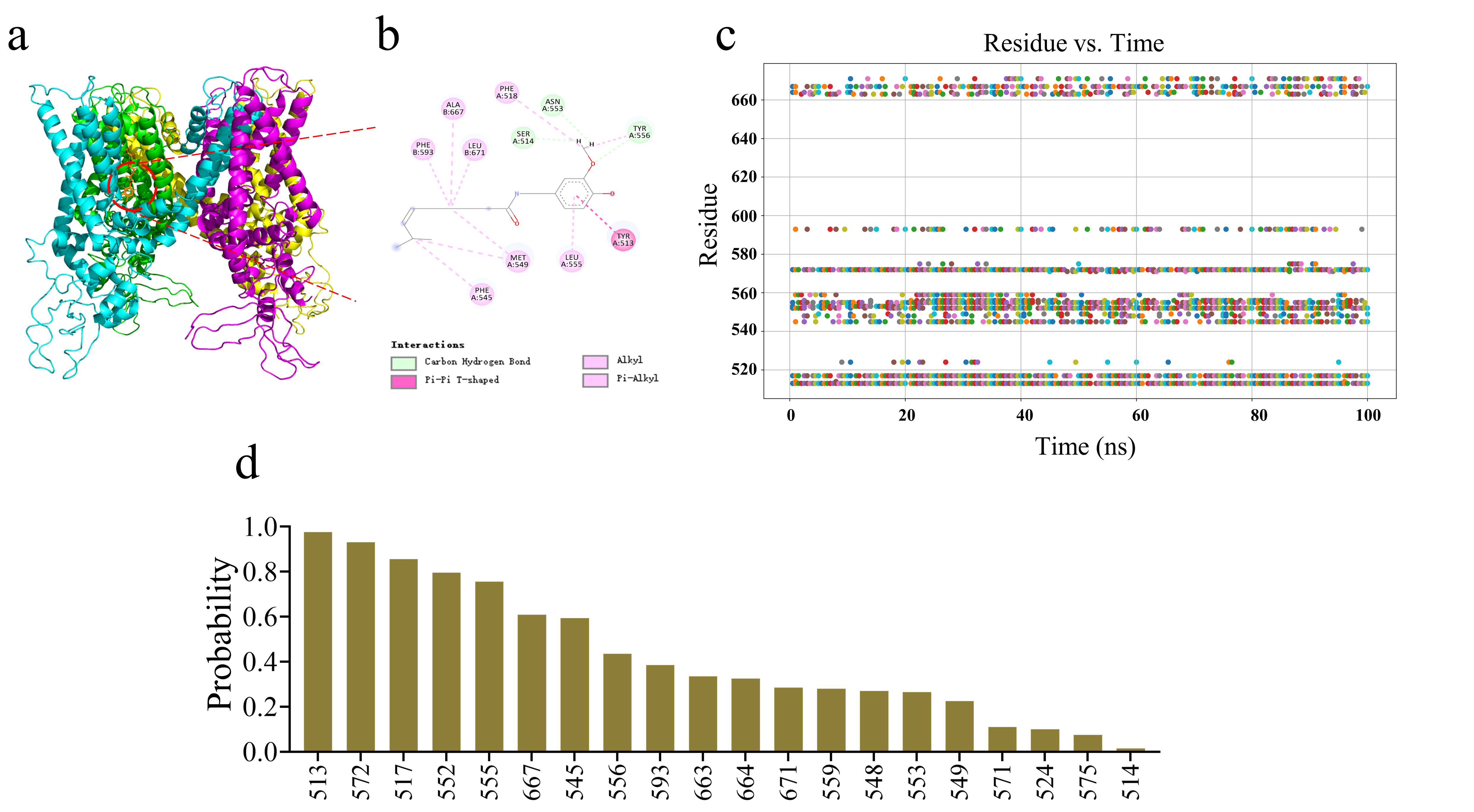


Figure S2. The molecular docking and molecular dynamics simulation between TRPV1 and capsaicine. (a-b) Visualization of the interactions between TRPV1 and capsaicine. (c) Examine the interactions between TRPV1 residues and capsaicine throughout a 100 ns simulation. The 100 ns trajectory was segmented into 200 frames, each representing the binding status of ligands to TRPV1 residues at every 0.5 ns. Distinct colors denote each 0.5 nanoseconds interval. (d) Analysis of the interaction probabilities between the TRPV1 residues and capsaicine.


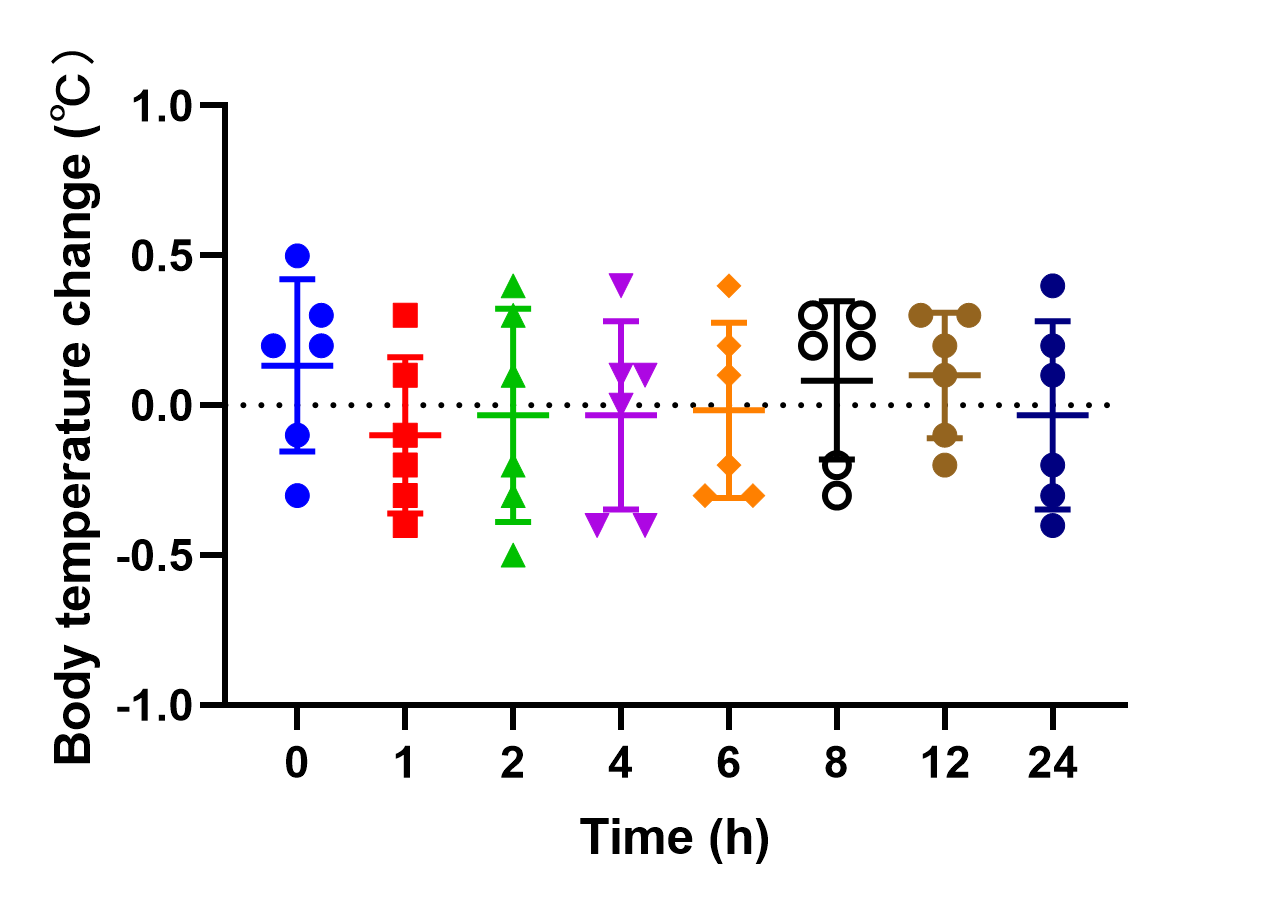
Figure S3. The body temperature change of mice after administration of physcion (20 mg/kg) (n = 6). Data are presented as mean ± SD.

Table S1 Primers for RT-PCR of DRG of mice

| Gene | Forward primer 5’-3’ | Reverse primer 5’-3’ |
| --- | --- | --- |
| *IL-1α* | CGAAGACTACAGTTCTGCCATT | GACGTTTCAGAGGTTCTCAGAG |
| *IL-1β* | GCAACTGTTCCTGAACTCAACT | ATCTTTTGGGGTCCGTCAACT |
| *IL-6* | TAGTCCTTCCTACCCCAATTTCC | TTGGTCCTTAGCCACTCCTTC |
| *IL-11* | GCGCTGTTCTCCTAACCCG | GAGTCCAGACTGTGATCTCCG |
| *CXCL10* | CCAAGTGCTGCCGTCATTTTC | GGCTCGCAGGGATGATTTCAA |
| *PKC* | CAGGGTATCTGGGGAATGGC | AGTCCATAATGAGAGGCAGGG |
| *CAMK2A* | TGGAGACTTTGAGTCCTACACG | CCGGGACCACAGGTTTTCA |
| *CAMK2B* | CGTTTCACCGACGAGTACCAG | GCGTACAATGTTGGAATGCTTC |
| *18S rRNA* | AGGAATTGACGGAAGGGCACCA | GTGCAGCCCCGGACATCTAAG |

Table S2 Primers for RT-PCR of RAW264.7

| Gene | Forward primer 5’-3’ | Reverse primer 5’-3’ |
| --- | --- | --- |
| *IL-1β* | GCAACTGTTCCTGAACTCAACT | ATCTTTTGGGGTCCGTCAACT |
| *IL-6* | TAGTCCTTCCTACCCCAATTTCC | TTGGTCCTTAGCCACTCCTTC |
| *IL-18* | GACTCTTGCGTCAACTTCAAGG | CAGGCTGTCTTTTGTCAACGA |
| *COX-2* | TTCAACACACTCTATCACTGGC | AGAAGCGTTTGCGGTACTCAT |
| *TGF-β* | CTCCCGTGGCTTCTAGTGC | GCCTTAGTTTGGACAGGATCTG |
| *TNF-α* | CCTGTAGCCCACGTCGTAG | GGGAGTAGACAAGGTACAACCC |
| *18S rRNA* | AGGAATTGACGGAAGGGCACCA | GTGCAGCCCCGGACATCTAAG |
